# Serine-Threonine Kinases Encoded by Split *hipA* Homologs Inhibit Tryptophanyl-tRNA Synthetase

**DOI:** 10.1128/mBio.01138-19

**Published:** 2019-06-18

**Authors:** Stine Vang Nielsen, Kathryn Jane Turnbull, Mohammad Roghanian, Rene Bærentsen, Maja Semanjski, Ditlev E. Brodersen, Boris Macek, Kenn Gerdes

**Affiliations:** a Centre for Bacterial Stress Response and Persistence, Section for Functional Genomics, Department of Biology, University of Copenhagen, Copenhagen, Denmark; b Centre for Bacterial Stress Response and Persistence, Department of Molecular Biology and Genetics, Aarhus University, Aarhus, Denmark; c Proteome Center Tübingen, Interfaculty Institute for Cell Biology, University of Tübingen, Tübingen, Germany; Massachusetts Institute of Technology; Imperial College London; Instituto de Tecnologia Quimica e Biologica-Universidade Nova de Lisboa

**Keywords:** persistence, ppGpp, toxin/antitoxin systems, translation, tRNA synthetase

## Abstract

Type II toxin-antitoxin (TA) modules encode a stable toxin that inhibits cell growth and an unstable protein antitoxin that neutralizes the toxin by direct protein-protein contact. *hipBA* of Escherichia coli strain K-12 codes for HipA, a serine-threonine kinase that phosphorylates and inhibits glutamyl-tRNA synthetase. Induction of *hipA* inhibits charging of glutamyl-tRNA that, in turn, inhibits translation and induces RelA-dependent (p)ppGpp synthesis and multidrug tolerance. Here, we describe the discovery of a three-component TA gene family that encodes toxin HipT, which exhibits sequence similarity with the C-terminal part of HipA. A genetic screening revealed that *trpS* in high copy numbers suppresses HipT-mediated growth inhibition. We show that HipT of E. coli O127 is a kinase that phosphorylates tryptophanyl-tRNA synthetase *in vitro* at a conserved serine residue. Consistently, induction of *hipT* inhibits cell growth and stimulates production of (p)ppGpp. The gene immediately upstream from *hipT*, called *hipS*, encodes a small protein that exhibits sequence similarity with the N terminus of HipA. HipT kinase was neutralized by cognate HipS *in vivo*, whereas the third component, HipB, encoded by the first gene of the operon, did not counteract HipT kinase activity. However, HipB augmented the ability of HipS to neutralize HipT. Analysis of two additional *hipBST*-homologous modules showed that, indeed, HipS functions as an antitoxin in these cases also. Thus, *hipBST* constitutes a novel family of tricomponent TA modules where *hipA* has been split into two genes, *hipS* and *hipT*, that function as a novel type of TA pair.

## Abstract (precis)

Bacterial toxin-antitoxin (TA) modules confer multidrug tolerance (persistence) that may contribute to the recalcitrance of chronic and recurrent infections. The first high-persister gene identified was *hipA* of Escherichia coli strain K-12, which encodes a kinase that inhibits glutamyl-tRNA synthetase. The *hipA* gene encodes the toxin of the *hipBA* TA module, while *hipB* encodes an antitoxin that counteracts HipA. Here, we describe a novel, widespread TA gene family, *hipBST*, that encodes HipT, which exhibits sequence similarity with the C terminus of HipA. HipT is a kinase that phosphorylates tryptophanyl-tRNA synthetase and thereby inhibits translation and induces the stringent response. Thus, this new TA gene family may contribute to the survival and spread of bacterial pathogens.

## INTRODUCTION

Prokaryotic toxin-antitoxin (TA) modules are usually composed of two elements, a toxin that can inhibit cell growth and an antitoxin that counteracts the inhibitory effect of the toxin (1, 2). Based on the molecular modes of antitoxin activity, TA modules have been divided into different types (3). The abundant type II modules are characterized by protein antitoxins that bind directly to and inhibit their cognate toxins by tight molecular interaction. Type II antitoxins usually contain a DNA-binding motif used to regulate TA operon transcription via binding to operators in the promoter region and a separate domain that interacts with and neutralizes the cognate toxin. Moreover, antitoxins are degraded by cellular proteases, such as Lon and/or Clp, and the cellular activity and amount synthesized of a given toxin are thus determined by the concentration of cognate antitoxin (4).

Type II modules are highly abundant; that is, most prokaryotic chromosomes encode at least one and some chromosomes encode cohorts of them. For example, Mycobacterium tuberculosis has 88 known, well-conserved type II TAs, while the insect pathogen Photorhabdus luminescens has a similarly large cohort (5). Toxin gene similarities were used to divide type II modules into superfamilies (6, 7). Thus, in general, toxins that exhibit sequence similarity inhibit cell growth by identical or related molecular mechanisms and can be grouped into the same family. Type II toxins belonging to the RelE, MazF, VapC, HipA, and TacT families curtail cell growth by inhibiting translation, CcdB and ParE inhibit DNA replication, Zeta toxins inhibit cell wall synthesis, and RES toxins inhibit cell growth by depleting NAD^+^ (8–18).

The biological functions of TAs have been debated. For type II modules, many studies now point to a function in survival during stress, including tolerance of multiple antibiotics (1). Stochastic or stress-induced activation of TA modules can protect bacteria from unfavorable environmental conditions by inducing persister formation (19, 20), a transient, slow-growing state in which the bacteria are tolerant of antibiotics and various other forms of stress (21). The stochastic formation of persisters is due to phenotypic heterogeneity in clonal populations of cells and can be viewed as a bet-hedging strategy that increases the survival rate in rapidly changing environments (22). Moreover, sublethal concentrations of antibiotics and other stresses have been found to stimulate the formation of persisters (23, 24).

The first gene associated with persistence was *hipA* (high persister gene A) of Escherichia coli strain K-12, identified as a gain-of-function allele, *hipA7* (25). This allele, found also in clinical isolates of uropathogenic E. coli (26), showed a 100- to 1,000-fold increase in persistence due to two amino acid changes in HipA (changes of G to S at position 22 [G22S] and D to A at positions 291 [D291A]) (27). The *hipA* toxin gene and the upstream *hipB* antitoxin gene together constitute a type II TA module (28). Modest ectopic expression of wild-type HipA causes severe growth inhibition that can be countered by the HipB antitoxin, which interacts directly with HipA (28). HipA and HipB form a complex that represses *hipBA* transcription via binding to operators in the promoter region (26). HipA is a Hanks serine-threonine kinase (29, 30) and was found to specifically phosphorylate and inhibit glutamyl-tRNA synthetase (GltX or GltRS), causing strong inhibition of translation and induction of guanosine tetra- and pentaphosphate [(p)ppGpp] synthesis and persistence (11, 31, 32). HipA-mediated phosphorylation of the conserved residue Ser239 inhibits the activity of GltX (11), thereby preventing charging of tRNA^Glu^. As a consequence, the ratio of charged to uncharged tRNA^Glu^ decreases, which in turn stimulates binding of RelA-tRNA complexes to the ribosome, leading to activation of RelA (33). The resulting increase in the cellular (p)ppGpp level triggers the stringent response (11, 27, 32).

**FIG 1:**
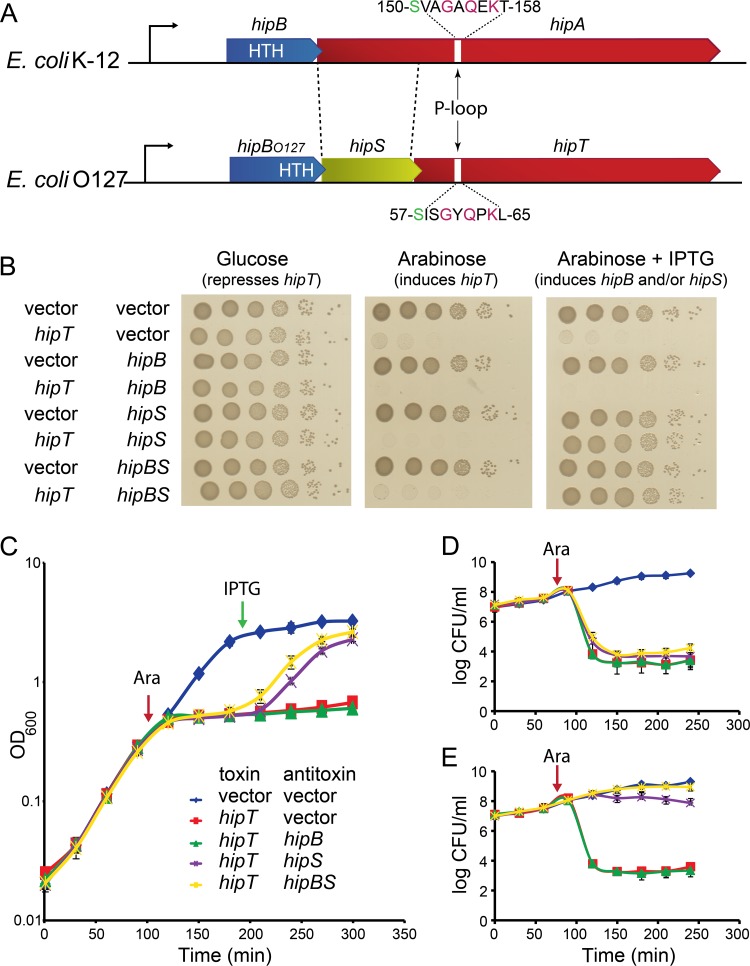
*hipBST* of E. coli O127 encodes a three-component toxin-antitoxin module. (A) Schematic showing a comparison of the *hipBA* and *hipBST* operons of E. coli K-12 and O127, respectively. Bent arrows pointing right indicate promoters. The *hipBA* operon contains two genes, *hipB* and *hipA*, while *hipBST_O127_* contains three genes, *hipB_O127_*, *hipS_O127_*, and *hipT_O127_*. The region of *hipA* between the dashed lines exhibits sequence similarity to *hipS_O127_*. The 8 amino acid residues of the P loop in HipA (150-VAGAQEKT-158) that binds phosphates of ATP are shown; the autophosphorylated S150 residue is shown in green (35). The homologous P loop and autophosphorylated serine in HipT_O127_ were inferred by sequence similarity. (B) Overnight cultures of E. coli MG1655 harboring pSVN1 (pBAD33::*hipT_O127_*) or the empty pBAD33 vector combined with pSVN111 (pNDM220::*hipB_O127_*), pSVN109 (pNDM220::*hipS_O127_*), pSVN110 (pNDM220::*hipBS_O127_*), or the empty low-copy-number pNDM220 vector, as indicated, were diluted to obtain the same values of OD_600_, centrifuged at 5,000 rpm for 5 min, washed in phosphate-buffered saline (PBS), and serially diluted before being spotted onto LB nutrient agar plates containing 0.2% glucose (to repress *hipT_O127_*), 0.2% arabinose (to induce *hipT_O127_*), or 0.2% arabinose plus 200 μM IPTG (to induce *hipB_O127_*, *hipS_O127_*, or *hipBS_O127_*). (C) The strains used in the experiment whose results are shown in panel B were grown in LB medium plus appropriate antibiotics. Overnight cultures were diluted, cells were grown exponentially for at least 3 h until the doubling time appeared constant, and at an OD_600_ of ≈0.3, arabinose (0.2%) was added to induce *hipT_O127_* (red arrow). After a further 1.5 h, IPTG (200 μM) was added to induce *hipS_O127_*, *hipB_O127_*, or *hipBS_O127_* (green arrow). (D and E) Viable counts of strains from the experiment whose results are shown in panels B and C before and after the addition of arabinose (0.2%) at an OD_600_ of ≈0.3 (red arrow). At each time point, cell samples (0.5 ml) were washed in PBS before a 10-times dilution series was spotted on agar plates with glucose (0.2%) to repress *hipT_O127_* expression (D) or with glucose (0.2%) to repress *hipT_O127_* expression and IPTG (200 μM) to induce *hipB_O127_*, *hipS_O127_*, or *hipBS_O127_* (E). Plates were incubated for 16 h at 37°C before counting. Data points in panels C, D, and E represent mean values of results from at least three independent experiments, and error bars indicate standard deviations.

Here, we describe a novel family of three-component TA modules encoding toxins exhibiting sequence similarity to HipA. We discovered that HipT of the enteropathogenic E. coli O127:H6 strain E2348/69 (HipT_O127_) is a toxin that can be counteracted by overproduction of tryptophanyl-tRNA synthetase (TrpS or TrpRS). Consistently, our *in vitro* data show that HipT_O127_ is a serine-threonine kinase that inhibits translation by phosphorylating TrpS. HipT_O127_ aligns colinearly with HipA but lacks ∼100 amino acids (aa) at its N terminus (Fig. 1A). Interestingly, *hipT_O127_* is preceded by *hipS_O127_*, encoding HipS_O127_ (103 aa), which exhibits sequence similarity with the N-terminal part of HipA that is missing from HipT_O127_ (Fig. 1A). Finally, *hipS_O127_* is preceded by a gene encoding a HipB homolog containing a helix-turn-helix (HTH) DNA-binding motif. HipB, HipS, and HipT form a complex *in vivo* and *in vitro*, and HipS_O127_ alone counteracts HipT_O127_ activity *in vivo*. The HipB homolog (called HipB_O127_) does not counteract HipT_O127_ but instead augments the ability of HipS_O127_ to counteract HipT_O127_. Analysis of the *hipBST* modules of Haemophilus influenzae and Tolumonas auensis revealed that the HipT proteins of these organisms also are counteracted by overproduction of TrpS. Moreover, cognate HipS neutralizes HipT in both these cases. In summary, we describe here a family of novel three-component TA modules that potentially can increase the stress resilience and spread of bacterial pathogens.

## RESULTS

### Homologs of HipA are encoded by three-gene operons.

Using similarity searching with HipA (440 amino acids [aa]) of E. coli K-12 as the query sequence, we identified a number of genes encoding HipA homologs that aligned colinearly with the C terminus of HipA but were shortened by ∼100 aa at their N termini (Fig. 1A, and see Fig. S1A in the supplemental material) (34). The HipA homologs contain P-loop motifs matching the experimentally validated P loop of HipA, as well as conserved catalytic domains and Mg^2+^ binding motifs, suggesting that, like HipA, HipT proteins are kinases (Fig. S1A) (35). A phylogenetic analysis showed that HipA and HipT group monophyletically in a cladogram based on 8 HipA and 40 HipT sequences (Fig. S1D) (36). The majority of the *hipT* genes were from gammaproteobacteria, but two HipT homologs deeply embedded in the phylogenetic tree were from a deltaproteobacterium and a firmicute (Streptococcus pneumoniae) (Fig. S1D). The HipT homolog from S. pneumoniae is identical to the HipT homolog of H. influenzae strain 10810 (Fig. S1D). These two organisms separated more than a billion years ago, and both are highly competent for DNA uptake and live in the same biological habitat (the upper respiratory tract). These observations raise the possibility that *hipBST* loci undergo lateral gene transfer between distantly related organisms.

10.1128/mBio.01138-19.1 FIG S1 Alignments of protein sequences encoded by *hipBST* loci from five different gammaproteobacteria and a phylogenetic analysis of HipT. (A) Alignment of HipT homologs with the C-terminal part of HipA of E. coli K-12. The 8-aa conserved P loop (35) in HipA indicated below the sequences was used as a fixed point to generate the alignment. The autophosphorylated Ser150 in HipA is fully conserved in the HipT homologs, and the HipT proteins contain conserved catalytic domains and Mg2^+^ binding motifs as indicated (34, 35, 38). The HipT sequences were from E. coli O127:H6 strain E2348/69 (GenBank accession number CAS11333.1), Haemophilus influenzae Rd KW20 (NCBI accession number NP_438824), Tolumonas auensis DSM 9187 (NCBI accession number WP_015879003.1), Vibrio cholerae O1 strain NHCM-017 (WP_050916634.1), and Vibrio halioticoli (GenBank accession number GAD88429.1). (B) Alignment of five HipS homologs encoded by genes just upstream of the genes encoding the HipT homologs shown in panel A with the N-terminal part of HipA. The HipS sequences were from Escherichia coli O127:H6 strain E2348/69 (GenBank accession number CAS11334.1), H. influenzae Rd KW20 (NCBI accession number NP_438825.1), Tolumonas auensis (NCBI accession number WP_015879002.1), V. cholerae O1 strain NHCM-017 (accession number WP_044125702.1), and Vibrio halioticoli (GenBank accession number GAD88428.1). (C) Alignment of five HipB homologs encoded by genes just upstream of the genes encoding the HipS homologs shown in panel B. The HipB sequences were from E. coli O127:H6 strain E2348/69 (GenBank accession number CAS11335.1), H. influenzae Rd KW20 (NCBI accession number NP_438826.1), Tolumonas auensis (NCBI accession number WP_083757795.1), V. cholerae O1 strain NHCM-017 (WP_050916568.1), and *V. halioticoli* (GenBank accession number AD88427.1). Color codes of the alignments are as follows: black on white, nonsimilar residues; blue on cyan, consensus residue derived from a block of similar residues at a given position; black on green, consensus residue derived from the occurrence of greater than 50% of a single, distinct residue; red on yellow, consensus residue derived from a completely conserved residue; and green on white, residue weakly similar to consensus residue. The alignments in panels A, B, and C were generated by using the commercial version of Vector NTI (Invitrogen). (D) Phylogenetic tree of 8 HipA and 40 HipT homologs. A sequence alignment of the proteins was accomplished using an online version of MUSCLE (36) provided by European Bioinformatics Institute at EMBL (https://www.ebi.ac.uk/Tools/msa/muscle/) and used to derive the phylogenetic tree presented as a cladogram. Sampling of the HipA and HipT sequences was accomplished using BLASTP at NCBI and was not exhaustive. Accession numbers of the proteins included in the cladogram were as follows: Photorhabdus luminescens TT01, CAE17272.1; Vibrio parahaemolyticus, WP_020904255.1; Vibrio cyclitrophicus, WP_102385597.1; Pantoea vagans, WP_095707190.1; Enterobacter ludwigii, WP_081112566.1; *Pantoea* GM01, EJL89497.1; E. coli K-12, P23874.2; Shigella dysenteriae, WP_119170656.1; Paraglaciecola psychrophila 170, AGH46782.1; Desulfobacula toluolica Tol2, CCK78946.1; *Marinobacter* LQ44, AMQ90498.1; *Alteromonas mediterranea* U7, AGP89851.1; *V. halioticoli* NBRC 102217, GAD88429.1; Tatumella ptyseos NCTC11468, SQK77238.1; Serratia plymuthica PRI-2C, ANS44223.1; Yersinia frederiksenii FDAARGOS 417, ATM86408.1; Pantoea agglomerans C410P1, AOE38443.1; Pantoea agglomerans L15, AZI51552.1; Pantoea agglomerans TH81, AYP22744.1; *T. auensis* DSM 9187, ACQ93535.1; E. coli MVAST0167, AML11457.1; E. coli O127:H6 E2348/69, CAS11333.1; *Buttiauxella* 3AFRM03, AYN26082.1; Pectobacterium atrosepticum 36A, ATY88941.1; Pectobacterium carotovorum 3-2, AVT56767.1; *P. carotovorum* 14A, AZK60881.1; Paraglaciecola polaris NIBIO1006, ASY75229.1; *P. polaris* NIBIO1392, ASY81571.1; *P. carotovorum* PCC21, AFR01461.1; *P. carotovorum* subsp. *brasiliense*, ARA78327.1; *Actinobacillus porcitonsillarum* 9953L55, AWI50429.1; H. influenzae NML-Hia-1, AOZ67195.1; H. influenzae R2866, ADO81720.1; H. influenzae F3031, CBY81777.1; H. influenzae 723, AJO91476.1; H. influenzae 10810, CBW28981.1; Streptococcus pneumoniae 2842STDY5881852, CVQ15586.1; H. influenzae 67P38H1, AVJ03036.1; H. influenzae FDAARGOS199, ARB90233.1; H. influenzae Rd KW20, NP_438824; Haemophilus aegyptius NCTC8502, SQH35811.1; H. influenzae F3047, CBY86290.1; H. influenzae NCTC8455 SQK92775.1; H. influenzae P650-8603, AXP41025.1; H. influenzae 86-028NP, AAX87695.1; H. influenzae C486, AJO89092.1; H. influenzae 84P36H1, AWP55634.1; and H. influenzae 5P28H1, AVI99364.1. Download FIG S1, PDF file, 2.2 MB. Copyright © 2019 Nielsen et al. 2019 Nielsen et al. This content is distributed under the terms of the Creative Commons Attribution 4.0 International license.

In all the *hipT*-containing organisms examined, we discovered short open reading frames adjacent to and upstream from *hipT*, which are herein called *hipS*, encoding proteins of ∼100 aa that exhibit sequence similarity to the missing N-terminal part of HipA (Fig. S1B). In all these cases, open reading frames upstream from *hipS* encode putative proteins of ∼100 aa containing HTH DNA-binding motifs (Fig. S1C). These putative HipB homologs may thus autoregulate the *hipBST* operons. We chose the *hipBST* module of E. coli O127:H6 strain E2348/69 as our primary model system for functional analysis (Fig. 1A). The *hipBST_O127_* module encodes HipB_O127_ (107 aa), HipS*_O127_* (103 aa), and HipT*_O127_* (335 aa). Gene pair *hipB_O127_* and *hipS_O127_* overlaps by 16 nucleotides (nt), and gene pair *hipS_O127_* and *hipT_O127_* overlaps by 1 nt, suggesting that the genes may be translationally coupled.

### HipT_O127_ inhibits cell growth and is counteracted by HipS_O127_.

We validated the components encoded by *hipBST_O127_* experimentally by inserting *hipT_O127_* into plasmid vector pBAD33 (carrying the arabinose-inducible pBAD promoter) and *hipS_O127_*, *hipB_O127_*, and *hipBS_O127_* into the low-copy-number R1 vector pNDM220 (carrying the synthetic, isopropyl-β-d-thiogalactopyranoside [IPTG]-inducible pA1/O4/O3 promoter) and subjected the standard E. coli K-12 strain MG1655 carrying combinations of these plasmids to growth assays and viable-count measurements. Induction of *hipT_O127_* resulted in strong inhibition of cell growth, both on plates and in liquid medium, supporting the hypothesis that HipT_O127_ can function as a toxin (Fig. 1B and C). Growth was rescued by induction of *hipS_O127_* alone but not by *hipB_O127_* alone, suggesting that HipS*_O127_* functions as the antitoxin (Fig. 1B and C). Coinduction of *hipB_O127_* and *hipS_O127_* provided a consistent, yet mild growth rescue advantage compared to the results for *hipS_O127_* alone, suggesting that HipB_O127_ augments the antitoxin activity of HipS_O127_ (Fig. 1C). Thus, HipB_O127_ does not function as a classical antitoxin.

Upon induction of *hipT_O127_*, CFU decreased dramatically (Fig. 1D). However, later induction of *hipS_O127_* or *hipS_O127_* plus *hipB_O127_* (by adding IPTG and glucose to the agar plates to induce P_A1/O4/O3_::*hipBS_O127_* and repress pBAD::*hipT_O127_*, respectively) fully rescued cell viability (Fig. 1E). This result showed that ectopic production of HipT*_O127_* induces a bacteriostatic condition from which the cells can be resuscitated. In support of this conclusion, strains in which *hipT_O127_* was induced recovered viability after prolonged incubation times (∼40 h), even in the absence of *hipS_O127_* or *hipBS_O127_* (Fig. S2).

10.1128/mBio.01138-19.2 FIG S2 Long-term incubation shows HipT of E. coli O127 induces bacteriostasis. Viable counts (CFU/ml) before and after *hipT_O127_* induction in the presence or absence of induction of *hipB_O127_* or *hipS_O127_* and *hipBS_O127_*. Cultures of E. coli MG1655 harboring pSVN1 (pBAD33::*hipT_O127_*) or the empty pBAD33 vector combined with pSVN111 (pND::*hipB_O127_*), pSVN109 (pND::*hipS_O127_*), pSVN110 (pND::*hipBS_O127_*), or the empty low-copy-number pND vector were growing exponentially in LB medium at 37°C. The pBAD promoter was induced by the addition of arabinose (0.2%) at an OD_600_ of ≈0.3 (red arrow). At each time point, 0.5-ml cell samples were washed in PBS before 10-times dilution series were spotted on agar plates with glucose only (0.2%), to repress *hipT_O127_* expression (A), or glucose plus IPTG (200 μM) to also induce *hipB_O127_* or *hipS_O127_* and *hipBS_O127_* (B) on the plates. Plates were incubated for 40 hours at 37°C before counting. Data points represent mean values from at least three independent experiments, and error bars indicate standard deviations. As seen by the results, the prolonged incubation period (16 h versus 40 h) allowed for the full recovery of viability, even in the absence of *hipS* or *hipBS* (compare Fig. S2 with Fig. 1D and E). Download FIG S2, PDF file, 0.4 MB. Copyright © 2019 Nielsen et al. 2019 Nielsen et al. This content is distributed under the terms of the Creative Commons Attribution 4.0 International license.

We were puzzled by the observation that HipS_O127_ but not HipB_O127_ exhibited antitoxin activity and therefore decided to analyze the *hipBST* modules of two additional gammaproteobacteria, Haemophilus influenzae Rd KW20 (*hipBST_Hi_*) and Tolumonas auensis DSM 9187 (*hipBST_Ta_*) (Fig. S1). Induction of *hipT_Hi_* or *hipT_Ta_* inhibited cell growth of E. coli MG1655 in liquid medium in both cases, and induction of cognate *hipS* genes was sufficient to neutralize the two HipT toxins (Fig. S3A and B). Like HipB_O127_, HipB_Hi_ augmented the ability of HipS_Hi_ to neutralize HipT_Hi_, as the presence of the HipBS_Hi_-encoding plasmid almost entirely prevented growth inhibition after induction of *hipT_Hi_* (Fig. S3A). HipB_Ta_ did not detectably augment the antitoxin effect of HipS_Ta_ in this experimental setup (Fig. S3B). We also tested *hipT* genes from strains of Vibrio cholerae and Vibrio halioticoli, but their induction was, for unknown reasons, not toxic in E. coli K-12 and the corresponding *hipBST* modules were not analyzed further.

10.1128/mBio.01138-19.3 FIG S3 HipT of H. influenzae and *T. auensis* inhibit cell growth and can be neutralized by cognate HipS. Cells were grown as described in the legend to Fig. 1C. As seen by the results, cognate HipB_Hi_ augments HipS_Hi_ in the neutralization of HipT_Hi_. Strains used in the experiment whose results are shown in panel A were E. coli MG1655 harboring pSVN135 (pBAD33::*hipT_Hi_*) or the empty pBAD33 vector combined with pSVN122 (pNDM220::*hipB_Hi_*), pSVN123 (pNDM220::*hipS_Hi_*), or pSVN139 (pNDM220::*hipBS_Hi_*) or the empty low-copy-number pNDM220 as indicated, and strains in the experiment whose results are shown in panel B were E. coli MG1655 harboring pSVN129 (pBAD33::*hipT_Ta_*) or the empty pBAD33 vector combined with pSVN126 (pNDM220::*hipB_Ta_*), pSVN127 (pNDM220::*hipS_Ta_*), or pSVN138 (pNDM220::*hipBS_Ta_*) or the empty low-copy-number pNDM220 as indicated. Data points represent mean values from at least two independent experiments, and error bars indicate standard deviations. Download FIG S3, PDF file, 0.3 MB. Copyright © 2019 Nielsen et al. 2019 Nielsen et al. This content is distributed under the terms of the Creative Commons Attribution 4.0 International license.

### HipB, HipS, and HipT form a ternary complex *in vivo*.

The above-described observations suggest that HipB_O127_, HipS_O127_, and HipT_O127_ might form a protein complex *in vivo*, as seen for other type II TA modules. To test this, we constructed a plasmid (pSVN94) encoding N-terminally His_6_-tobacco etch virus (TEV)-tagged HipB_O127_, HipS_O127_, and the enzymatically inactive HipT_O127_^D233Q^ mutant protein in which all three genes had optimized translation signals (Shine-Dalgarno [SD] sequences and ATG start codons) to increase translation rates. His_6_-TEV-HipB_O127_ was purified under native conditions and analyzed by denaturing polyacrylamide gel electrophoresis (SDS-PAGE). Indeed, three proteins of the expected molecular weights (MWs) copurified (Fig. S4A), indicating that the HipBST_O127_ proteins form a complex *in vivo*. Further separation of the protein complex using a heparin column allowed isolation of three samples containing HipT_O127_, HipBT_O127_, and HipBST_O127_ (Fig. S4B, top). Gel filtration chromatography further confirmed that HipT and HipBST are monodispersed in solution, suggesting that HipBST_O127_ is a heterotrimer (Fig. S4B, bottom).

10.1128/mBio.01138-19.4 FIG S4 HipB_O127_, HipS_O127_, and HipT_O127_ form a complex. (A) Purification of His_6_-TEV-HipB_O127_, HipS_O127_, and HipT^D233Q^_O127_ from E. coli strain BL21 containing pSVN94 analyzed by SDS-PAGE. E. coli BL21 containing pSVN94 was grown in LB medium at 37°C and His_6_-TEV-HipB_O127_ purified according to standard procedure. As seen by the results, the N-terminally His-tagged HipB_O127_ pulled down HipS_O127_ and HipT_O127_. Lanes 1 to 5 contained samples from washes with buffer B (50 mM NaH_2_PO_4_ [pH 8], 0.3 M NaCl, 35 mM imidazole, and 1 mM β-mercaptoethanol). Lanes 6 to 10 show samples from washes with buffer C (100 mM NaH_2_PO_4_ [pH 8], 10 mM Tris-HCl [pH 8], and 1 mM β-mercaptoethanol) with different concentrations of urea, as follows: 0 M, 2.45 M, 4.9 M, 7.35 M, and 9.8 M urea, respectively. Lane 11 shows a sample from flowthrough after overnight wash with buffer C containing 9.8 M urea. Lanes 12 and 13 show samples from additional washes with buffer C containing 9.8 M urea after overnight incubation. Lane 14 contained a sample from elution with buffer D (100 mM NaH_2_PO_4_ [pH 8], 10 mM Tris-HCl [pH 8], 9.8 M urea, 0.5 M imidazole, and 1 mM β-mercaptoethanol). (B) Top, purification of HipBST_O127_ subcomplexes using a heparin column and elution using increasing concentrations of salt. Bottom, purification of HipBST_O127_ and isolated HipT_O127_ toxin on a gel filtration column. Elution positions of standard proteins with known mass (indicated in kDa) are shown with vertical arrows. Download FIG S4, PDF file, 2.9 MB. Copyright © 2019 Nielsen et al. 2019 Nielsen et al. This content is distributed under the terms of the Creative Commons Attribution 4.0 International license.

### Multicopy suppression of HipT by *trpS*.

**FIG 2:**
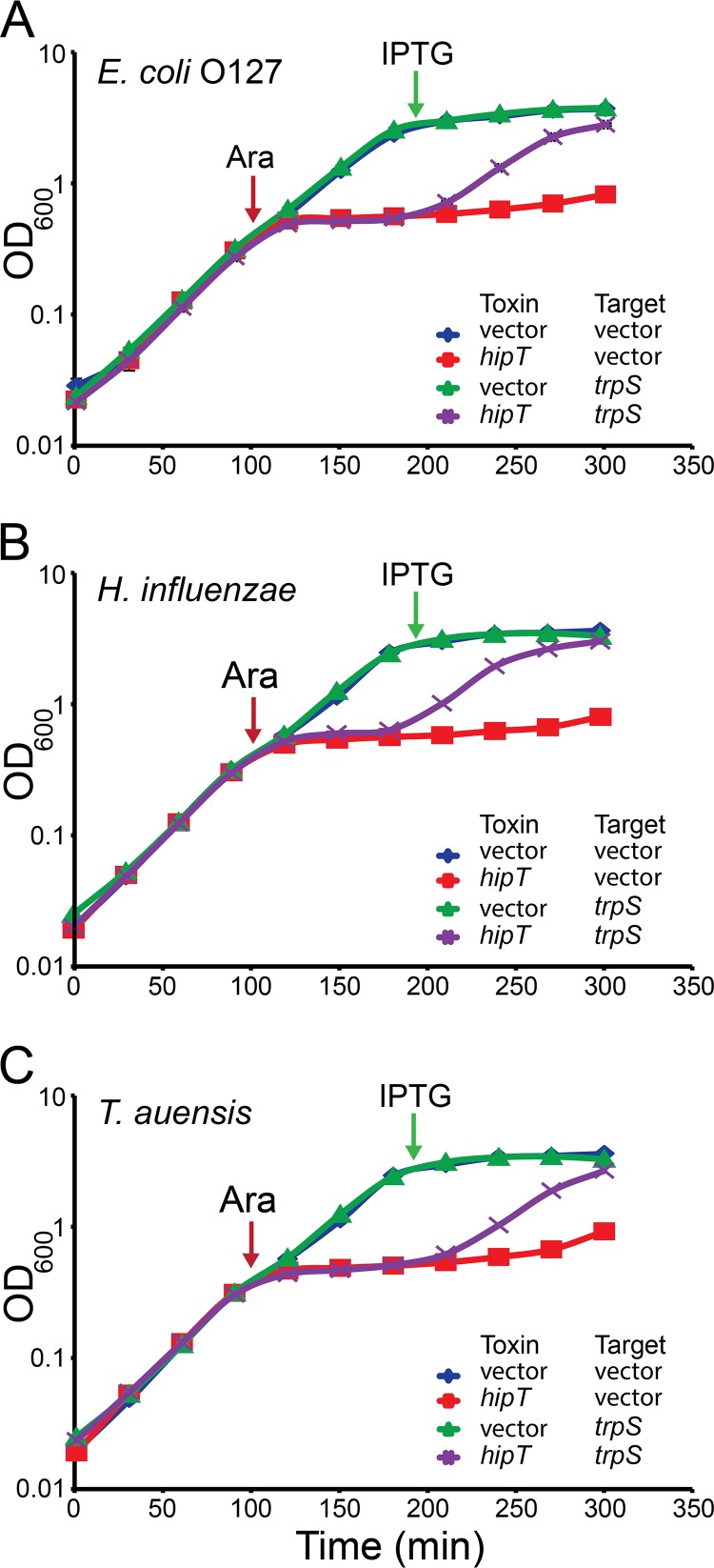
Overproduction of TrpS counteracts HipT_O127_. (A to C) Growth curves of strains of E. coli MG1655 harboring pSVN1 (pBAD33::*hipT_O127_*) (A), pSVN135 (pBAD33::*hipT_Hi_*) (B), and pSVN129 (pBAD33::*hipT_Ta_*) (C) or the empty pBAD33 vector combined with pSVN37 (pEG25::*trpS*) or the empty high-copy-number pEG25 vector, as indicated. Cells were grown in LB medium supplemented with the appropriate antibiotics. Overnight cultures were diluted and grown exponentially for at least 3 h until the doubling time appeared constant. The pBAD promoter of the pBAD33 derivatives was induced by arabinose (0.2%) at an OD_600_ of ≈0.3 (red arrow). The P_A1/O4/O3_ promoter of the pEG25-derived plasmids was induced by the addition of IPTG (200 μM; green arrow) 1.5 h later. Data points represent mean values from at least two independent experiments, and error bars indicate standard deviations.

Previously, we showed that overproduction of GltX suppresses HipA-mediated growth inhibition and that HipA phosphorylates GltX *in vitro* (11). Unexpectedly, overproduction of GltX did not suppress HipT-mediated growth inhibition (Fig. S5A). Therefore, we performed a second multicopy gene library screening in an attempt to identify genes that in high copy numbers could suppress the effect of HipT_O127_ (see Materials and Methods). Using a pBR322-based Sau3A-derived gene library of E. coli MG1655Δ*ydeA*, ∼8,300 colonies with an average insert size of ∼3,300 bp were screened, resulting in a coverage of roughly 5.8 times. In this screening, 105 hits were obtained, of which 19 were retransformed. Six of these plasmids exhibited a stable phenotype and were sent for sequencing. Thereby, we identified a DNA fragment containing *rpe*, *gph*, and *trpS* that suppressed HipT_O127_. Of these genes, only conditional induction of *trpS*, which encodes tryptophanyl-tRNA synthetase (TrpS), suppressed HipT_O127_-mediated growth inhibition, both on solid medium (Fig. S5B) and in liquid culture (Fig. 2A). TrpS also suppressed HipT_Hi_ and HipT_Ta_ (Fig. 2B and C and Fig. S5B), whereas GltX had no such effect (Fig. S5A).

10.1128/mBio.01138-19.5 FIG S5 Overproduction of TrpS suppresses HipT_O127_, HipT_Hi_, and HipT_Ta_-mediated growth inhibition, whereas GltX has no such effect. (A) Overnight cultures of E. coli MG1655 harboring (left) pSVN1 (pBAD33::*hipT_O127_*), (middle) pSVN135 (pBAD33::*hipT_Hi_*), or (right) pSVN129 (pBAD33::*hipT_Ta_*) or the empty pBAD33 vector combined with pEG::*gltX* (pMG25::*gltX*) or the empty high-copy-number vector pMG25, as indicated, were diluted to similar OD_600_ values and washed in PBS before 10-times dilution series were spotted on LB agar plates containing the appropriate antibiotics and glucose (0.2%), arabinose (0.2%), or arabinose (0.2%) plus IPTG (200 μM), respectively. (B) Overnight cultures of E. coli MG1655 harboring (left) pSVN1 (pBAD33::*hipT_O127_*), (middle) pSVN135 (pBAD33::*hipT_Hi_*), or (right) pSVN129 (pBAD33::*hipT_Ta_*) or the empty pBAD33 vector combined with pSVN103 (pNDM220::*trpS*) or the empty low-copy-number vector pNDM220, as indicated, were diluted to similar OD_600_ values and washed in PBS before 10-times dilution series were spotted on LB agar plates containing the appropriate antibiotics and glucose (0.2%), arabinose (0.2%), or arabinose (0.2%) plus IPTG (200 μM), respectively. Download FIG S5, PDF file, 0.2 MB. Copyright © 2019 Nielsen et al. 2019 Nielsen et al. This content is distributed under the terms of the Creative Commons Attribution 4.0 International license.

### HipT phosphorylates TrpS at a conserved sequence motif.

**FIG 3:**
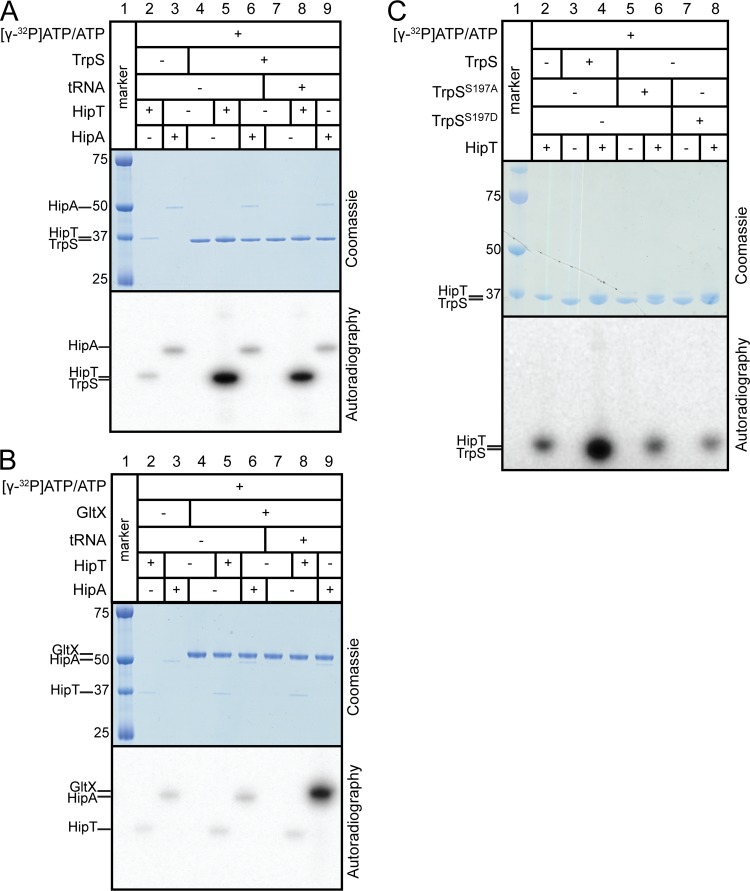
HipT_O127_ phosphorylates TrpS at S197 *in vitro*, and HipT_O127_ is autophosphorylated *in vitro*. (A) Phosphorylation of TrpS and autophosphorylation of HipT_O127_ and HipA *in vitro*. HipT_O127_ or HipA (0.2 μM), [γ-^32^P]ATP (0.1 μM), and ATP (66 μM) were incubated with (+) or without (−) TrpS [purified from strain C41(DE3)/pSVN46], as well as with (+) or without (−) a mixture of all E. coli tRNAs (0.5 μg) per microliter of reaction mixture as indicated. (B) Phosphorylation of GltX and autophosphorylation of HipT_O127_ and HipA *in vitro*. HipT_O127_ or HipA (0.2 μM), [γ-^32^P]ATP (0.1 μM), and ATP (66 μM) were incubated with (+) or without (−) GltX (purified from strain JW2395), as well as with (+) or without (−) a mixture of E. coli tRNAs (0.5 μg) per microliter of reaction mixture as indicated. (C) Phosphorylation of TrpS at S197 and autophosphorylation of HipT_O127_ *in vitro*. HipT_O127_ (1.5 μM) [purified from strain C41(DE3)/pSVN42] was added to the reaction mixtures as indicated, in addition to [γ-^32^P]ATP (0.1 μM) and ATP (66 μM) with (+) or without (−)TrpS (1.5 μM), TrpS^S197A^ (1.5 μM), or TrpS^S197D^ (1.5 μM) as indicated.

The above-described results suggested that HipT phosphorylates TrpS. To analyze HipT kinase activity directly, we purified HipT_O127_ and its presumed target, TrpS. For comparison, we included HipA and its known target GltX in the analysis. Indeed, HipT_O127_ phosphorylated TrpS *in vitro* (Fig. 3A, lanes 5 and 8) in a reaction that did not require tRNA (Fig. S6). We showed previously that HipA phosphorylates GltX *in vitro* in a reaction that requires the addition of tRNA^Glu^ (11). Here, we were able to reproduce the results showing that HipA phosphorylates GltX in the presence but not in the absence of tRNA (Fig. 3B, lanes 6 and 9). Thus, HipT and HipA kinases differ not only with respect to their specific target but also by whether there is a requirement for the presence of tRNA in the *in vitro* reaction mixtures (see Discussion).

10.1128/mBio.01138-19.6 FIG S6 tRNA is not required for *in vitro* phosphorylation of TrpS by HipT_O127_. Purified TrpS (1 μM; purified from E. coli BL21/pSVN46), HipT_O127_ (0.5 μM) (purified from E. coli BL21/pSVN42), and RNase A (0.1 mg/ml) were mixed with 0.1 μM [γ-^32^P]ATP and 66 μM ATP as indicated. Samples were treated as described in Materials and Methods. Download FIG S6, PDF file, 1.1 MB. Copyright © 2019 Nielsen et al. 2019 Nielsen et al. This content is distributed under the terms of the Creative Commons Attribution 4.0 International license.

The best-conserved stretch of amino acids between GltX and TrpS are the highly conserved KLS^239^KR/KMS^197^KS flexible-loop motifs (Fig. S7). Lys237 and Lys195 participate in the catalytic reaction by stabilizing the transition state of ATP, and intact loop motifs are required for catalysis (37). The observation that HipA phosphorylates GltX at S239 (11) raised the possibility that HipT phosphorylates TrpS at the homologous S197. To test this, we introduced two amino acid changes, S197A and S197D, into TrpS, the latter to mimic a phosphorylated serine. Both changes abolished phosphorylation of HipT_O127_, consistent with the proposal that HipT phosphorylates TrpS at S197 (Fig. 3C). Finally, mass-spectrometric analysis revealed that, indeed, HipT_O127_ phosphorylates TrpS at S197 *in vitro* (Table 1). We also note that HipA did not phosphorylate TrpS (Fig. 3A, lanes 6 and 9), while HipT_O127_ did not phosphorylate GltX (Fig. 3B, lanes 5 and 8). This lack of cross-reactivity in the *in vitro* reactions is consistent with the specificity of the multicopy suppression data.

**TABLE 1:** Phosphorylation sites identified by LC-MS/MS analysis of products of *in vitro* phosphorylation reaction between HipT_O127_ and TrpS

Protein	Amino acid	Andromeda score	Localization probability	Mass error (ppm)	Phosphopeptide sequence of the best localized MS/MS spectrum
HipT	S57	104.24	0.999992	0.93417	GMS(1)ISGYQPK
HipT	S59	139.32	0.999224	0.14379	GMS(0.001)IS(0.999)GYQPK
TrpS	S197	304.44	0.999903	−0.17001	KMS(1)KSDDNRNNVIGLLEDPK
TrpS	S199	309.72	0.864936	−0.094932	SGARVMSLLEPTKKMS(0.135)KS(0.865)DDNRNNVIGLLEDPK

10.1128/mBio.01138-19.7 FIG S7 Sequence alignment of GltX and TrpS. The alignment shows that S239 in GltX aligns to S197 in TrpS in the conserved sequence motifs KKLSKR and KKMSKS in GltX and TrpS, respectively. Download FIG S7, PDF file, 0.4 MB. Copyright © 2019 Nielsen et al. 2019 Nielsen et al. This content is distributed under the terms of the Creative Commons Attribution 4.0 International license.

HipA is known to inactivate itself by *trans* autophosphorylation at Ser150 (35, 38). In the reaction mixture containing only HipT_O127_, a faint radioactive band corresponding to the MW of HipT_O127_ was observed (Fig. 3A and B, lane 2). Since HipT_O127_ was the only protein in the reaction mixture, we infer that HipT_O127_ phosphorylates itself. Consistently, the weak HipT_O127_ band also appeared when HipT_O127_ was mixed with the noncognate target GltX (Fig. 3B, lanes 5 and 8). Accordingly, the analysis of the products of the *in vitro* reaction between HipT_O127_ and TrpS by mass spectrometry showed that HipT_O127_ autophosphorylates either on S57 or S59 (Table 1).

### HipT_O127_ stimulates production of (p)ppGpp.

**FIG 4:**
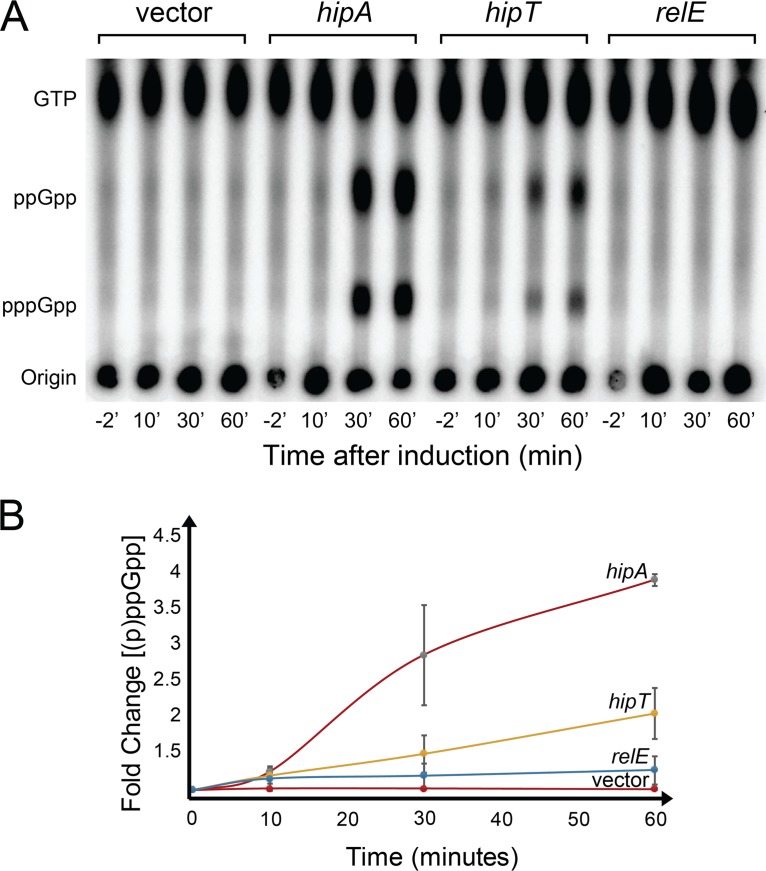
HipT_O127_ induces (p)ppGpp accumulation *in vivo*. Levels of (p)ppGpp of E. coli MG1655 containing pNDM220 (vector), pAH1 (pNDM220::*hipA*), pSVN116 (pNDM220::*hipT_O127_*), or pAH2 (pNDM220::*relE*). The toxin-encoding genes were located downstream from the IPTG-inducible P_A1/O3/O4_ promoter (56, 57). (A) Cells were grown exponentially at 37°C in low-phosphate MOPS (morpholinepropanesulfonic acid) minimal medium containing radiolabeled H_3_^32^PO_4_ (see Materials and Methods). Samples were withdrawn before and 10, 30, and 60 min after the addition of IPTG (1 mM) and analyzed by thin-layer chromatography (TLC) and phosphor imaging. (B) Quantification of the results of experiment shown in panel A and of repetitions of the experiment shown in Fig. S8. Materials and Methods gives additional experimental details. Error bars indicate standard deviations of three independent experiments.

We and others showed previously that HipA activates RelA to synthesize (p)ppGpp (11, 31, 32). Here, we measured whether induction of *hipT_O127_* induces (p)ppGpp synthesis and compared it to the effect of induction of *hipA* or *relE* of E. coli K-12, the latter of which inhibits translation by ribosome-dependent mRNA cleavage (8). Indeed, induction of both *hipT_O127_* and *hipA* resulted in increased levels of (p)ppGpp, albeit at a somewhat lower level in the case of *hipT_O127_* (Fig. 4A and B). The latter observation is consistent with the fact that tryptophan is encoded by one codon only, compared to two in the case of glutamate, and the fraction of tryptophanyl-tRNA is less than 2% of total tRNA, whereas that of glutamyl-tRNA is more than 7% (39). Thus, deficiency of charged tRNA^Glu^ leads to a higher level of hungry ribosomal A sites and, therefore, a higher number of activated RelA molecules and a higher level of (p)ppGpp. Consistent with previous results (40), induction of *relE* did not stimulate (p)ppGpp synthesis, showing that inhibition of translation *per se* is not sufficient to stimulate (p)ppGpp accumulation (Fig. 4A and B).

10.1128/mBio.01138-19.8 FIG S8 (p)ppGpp accumulation following overproduction of HipA, HipT_O127_, and RelE. The figure shows a repetition of the experiment whose results are shown in Fig. 4A. Cells of E. coli MG1655 carrying pAH1 (pNDM220::*hipA*), pSVN116 (pNDM220::*hipTO127*), or pAH2 (pNDM220::*relE*) were grown exponentially in low-phosphate MOPS minimal medium containing H^32^PO_4_. Samples were withdrawn before and 10, 30, and 60 minutes after induction of the toxin genes by the addition of IPTG (1 mM) and analyzed by TLC and phosphor imaging. The data contributed to the quantification shown in Fig. 4B. Materials and Methods gives additional experimental details. Download FIG S8, PDF file, 2.7 MB. Copyright © 2019 Nielsen et al. 2019 Nielsen et al. This content is distributed under the terms of the Creative Commons Attribution 4.0 International license.

## DISCUSSION

In this paper, we describe the discovery of a novel family of bacterial serine/threonine kinases, HipT kinases, that exhibit sequence similarity with HipA of E. coli K-12. HipA inhibits GltX (glutamyl-tRNA synthetase) by phosphorylation and thereby triggers RelA-dependent synthesis of (p)ppGpp (11, 31, 32). We found that HipT of E. coli O127 phosphorylates and inhibits TrpS (tryptophanyl-tRNA synthetase) and thereby, similarly to HipA, stimulates synthesis of (p)ppGpp (Fig. 4). Even though TrpS and GltX belong to the same class of tRNA synthetases (41), HipT_O127_ and HipA do not exhibit cross-phosphorylation of TrpS and GltX *in vitro*, implying that the two kinases exhibit substrate specificity (Fig. 3A and B). We showed previously that HipA phosphorylates S239 of the conserved KLS^239^KR motif in GltX (11). A variant of this motif (KMS^197^KS) is present in TrpS. Even though there are two amino acid differences between the two motifs, they represent the overall highest degree of sequence similarity between the two synthetases, suggesting that HipT phosphorylates S197 of TrpS. Indeed, this proposal was confirmed by our mass spectrometric and mutational analysis of TrpS (Table 1 and Fig. 3C).

We showed previously that phosphorylation of the conserved S239 of GltX by HipA requires the presence of tRNA^Glu^ in the *in vitro* reaction mixture (11). We proposed that the binding of tRNA^Glu^ to GltX would induce a conformational change of the motif KLS^239^KR that would make S239 accessible to phosphorylation (11). In contrast, even though GltX and TrpS belong to the same class of tRNA synthetases and the structural organization of their active sites is similar (42), phosphorylation of TrpS by HipT_O127_ does not require the addition of tRNA (Fig. S6 in the supplemental material). We believe that this difference is consistent with the requirement of GltX for the presence of cognate tRNA to activate glutamate to glutamyl-adenylate (41), a property shared with only two other type I tRNA synthetases (GlnRS and ArgRS). Thus, TrpS does not require the presence of tRNA^Trp^ to activate tryptophan to tryptophanyl-adenylate and does not require tRNA^Trp^ to be phosphorylated by HipT (Fig. S6).

HipA inactivates itself by autophosphorylation at the fully conserved, essential S150 located adjacent to the P loop of the kinase (35). Structural analysis revealed that autophosphorylation stabilizes a conformation of HipA that disrupts the ATP-binding pocket. It was proposed that autophosphorylation of HipA functions in the resuscitation of cells inhibited by HipA by preventing further activity of available toxins. This explanation is plausible, because cells inhibited by HipA somehow must revert the inhibition of GltX before the cells can resuscitate. We observed that HipT_O127_ is autophosphorylated *in vitro* (Fig. 3A and B) at the fully conserved S57 adjacent to the P-loop motif in HipT and, to a minor extent, at S59 in the P-loop motif, both of which are likely to inactivate the enzyme (Table 1).

**FIG 5:**
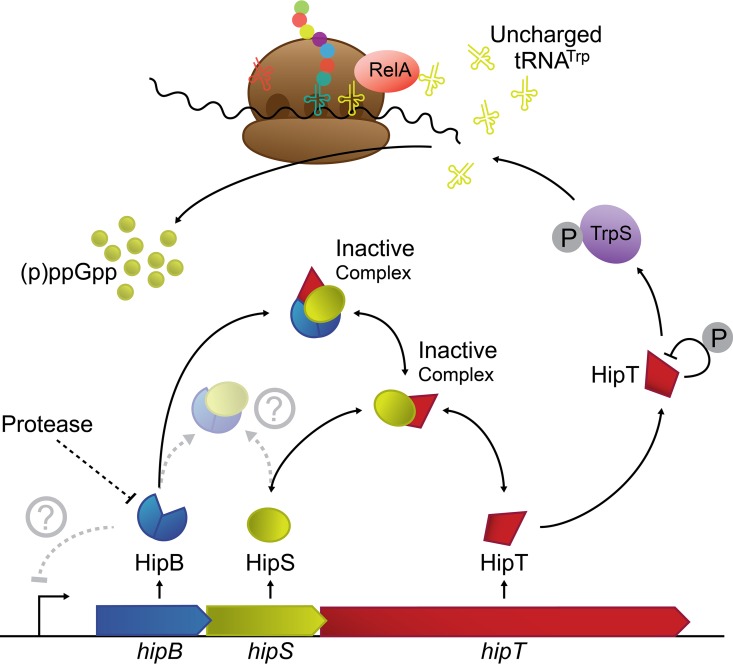
Schematic overview of the components encoded by *hipBST* and their interaction. Our evidence supports the idea that HipT inactivates TrpS by phosphorylation and that HipT phosphorylates itself. Inactivation of TrpS, in turn, increases the level of uncharged tRNA^Trp^ that, in complex with RelA, loads at hungry ribosomal A sites loaded with tryptophan codons. Loading of the binary RelA-tRNA^Trp^ complex at an A site activates RelA to synthesize (p)ppGpp (33). The function of HipT autophosphorylation is unknown, but it may play a role in the resuscitation of HipT-induced persister cells. Furthermore, our data show that the HipBST proteins form one or more complexes and that HipT is inactivated by HipS. HipB contains an HTH DNA-binding motif and probably autoregulates the *hipBST* operon. Speculative interactions are indicated by stippled lines.

The *hipT* gene is the third gene of the *hipBST* operon, and HipS and HipT exhibit sequence similarity with either end of HipA. The most parsimonious explanation as to how this arrangement appeared seems to be that *hipA* was duplicated during evolution and split into *hipS* and *hipT*, shifting the kinase specificity during this evolutionary trajectory. Analysis of the structure of HipBA reveals that HipS likely corresponds to the N-terminal subdomain of HipA, which was found to be involved in dimerization during DNA binding, as well as to harbor several mutations associated with high-persister phenotypes (Fig. S9A and B, blue) (26). A more detailed look at the N-terminal subdomain of HipA shows that residues involved in forming the hydrophobic core of the domain are well conserved in HipS, suggesting that HipS and the N-terminal subdomain of HipA share structure, while residues that are involved in HipA-HipA dimerization appear to differ in HipS while being conserved between HipS orthologs. This could suggest that the higher-order structure of HipBST differs from that of HipBA. We also note that several of the known high-persister mutations found in the N-terminal subdomain of HipA (including one of the mutations responsible for the *hipA7* genotype) are naturally present in HipS, which raises the possibility that HipS is HipA7-like (Fig. S9C). Finally, the structural analysis also reveals that HipB (of HipBST) closely matches the corresponding antitoxin HipB in HipBA and likely harbors a DNA-binding domain (Fig. S9A and B). Of the three proteins, the function of HipS as the “third TA component” is clearly the most intriguing. We found that all three HipS orthologs investigated are able to counteract cognate HipT toxins on their own, while the HipB proteins do not have such an effect (Fig. 1). However, in two cases, we observed that the HipB proteins augmented HipS-mediated neutralization of HipT, suggesting that HipB somehow increases the activity of HipS, for example, by increasing HipS metabolic stability or by stabilizing the HipS-HipT interaction. The latter proposal is consistent with the observation that HipBST form a stable complex *in vivo* (Fig. S4). A summary of our findings is presented in Fig. 5 and described further in the legend to the figure.

10.1128/mBio.01138-19.9 FIG S9 Modelling of the HipBST_O127_ complex structure. (A). Top, the crystal structure of E. coli HipBA (PDB ID 4YG7) colored according to a sequence alignment with HipBST_O127_, with the part corresponding to HipB_O127_ in purple, HipS_O127_ in blue, and HipT_O127_ in yellow. Bottom, schematic overview of the sequence alignment with colors as described above. HipA of E. coli K-12 is shown in red. (B) The structure of HipBA bound to DNA shows that the part corresponding to HipS_O127_ (the HipA N-terminal subdomain) mediates complex dimerization in the DNA-bound form. (C) The N-terminal subdomain of HipA contains a hydrophobic core conserved in HipS_O127_ (green) but a divergent dimerization interface (red). Two high-persister mutations in the N-subdomain-1 of HipA conserved among HipS orthologs are shown in yellow. Download FIG S9, PDF file, 0.2 MB. Copyright © 2019 Nielsen et al. 2019 Nielsen et al. This content is distributed under the terms of the Creative Commons Attribution 4.0 International license.

Although two-component TA modules are by far the most common, a number of other three-component TA modules have been identified (1). In many of these cases, two adjacent genes exhibit sequence similarity with known type II TA modules, while the function of the third component often remains unclear. However, in a few cases, the function of the third component is known. For example, M. tuberculosis contains a three-gene TA module that encodes a RelE-homologous HigB toxin and the HigA antitoxin. The third gene encodes a SecB-like chaperone that controls the stability of HigA such that the antitoxin becomes metabolically unstable under environmental stress, thereby leading to activation of HigB and inhibition of translation by mRNA cleavage (43). Thus, in this TA module, the third component provides a link between cellular physiology and activation of the TA module. ω-ε-ζ of Streptococcus pyogenes is a three-component TA module in which ω is a DNA-binding autorepressor of the operon and ε is an antitoxin that neutralizes the ζ toxin by direct protein-protein contact (44). In the *paaR-paaA*-*parE* modules of E. coli O157:H7, the first gene encodes a transcriptional regulator of the module operon and the second a type II antitoxin that counteracts the activity of the ParE toxin (45). Thus, our work presented here reveals a novel type of three-component TA modules with unknown regulator properties that will be important and exciting to study. We hope that future biochemical and structural studies will be helpful in revealing the mechanisms of HipBST activation and regulation, as well as why this locus is configured as a three-component TA module.

## MATERIALS AND METHODS

### Strains, plasmids, media, and growth conditions.

Strains and plasmids are listed in Table 2, and DNA oligonucleotides in Table 3. Cultures were grown at 37°C with shaking at 160 rpm in Luria-Bertani (LB) medium. When required, the medium was supplemented with 25 μg/ml chloramphenicol, 30 μg/ml or 100 μg/ml ampicillin, and 25 μg/ml kanamycin. Gene expression from plasmids carrying the pBAD promoter was induced by 0.2% arabinose and repressed by 0.2% glucose. Gene expression from plasmids carrying the synthetic P_A1/O4/O3_ promoter was induced by 200 μM isopropyl β-d-1 thiogalactopyranoside (IPTG). The solid medium used to grow cells was Luria-Bertani agar (LB agar) medium supplemented with the appropriate antibiotics and incubated at 37°C for approximately 16 h unless otherwise stated.

**TABLE 2:** Bacterial strains and plasmids

Strain or plasmid	Description^*a*^	Reference or source
E. coli strains		
MG1655	Wild-type K-12	58
MG1655*ΔydeA*	K-12 MG1655*ΔydeA*::FRT	This work
BL21	F^−^ *ompT hsdS*_B_ (r_B_^−^ m_B_^−^) *gal dcm*	59
C41 (DE3)	Derived from BL21(DE3): F^−^ *ompT hsdS*_B_ (r_B_^−^ m_B_^−^) *gal dcm* (DE3)	60
EG235	C41 (DE3) *ΔhipBA*::*kan*, pMG25::*gltX* (optimized SD), pBAD33::His_6_::*hipA* (SD8 and start codon ATG)	Laboratory collection
JW2395	AG1 [*recA1 endA1 gyrA96 thi-1 hsdR17 supE44 relA1*] carrying pCA24N::*gltX*, GltX purification plasmid encoding N-terminally His_6_-tagged *gltX*, from ASKA collection	55
Plasmids		
pBAD33	p15 *araC* P_BAD_, Cm^r^	61
pNDM220	Mini-R1 *lacI*^q^ P_A1/04/03_, Amp^r^	56
pCP20	pSC101 *rep*(Ts), Amp^r^ Cm^r^	47
pBR322	pMB1 *rop*, Amp^r^ Tet^r^	62
pMG25	pUC *lacI*^q^ P_A1/O4/O3_, Amp^r^	Laboratory collection
pEG25	pMG25 derivative that has reduced leakiness of the IPTG-inducible P_A1/O4/O3_ promoter	Laboratory collection
pEG::*gltX*	pMG25::*gltX*, optimized SD	Laboratory collection
pEG::_His6_*hipA*	pBAD33::_His6_*hipA*, HipA purification plasmid harboring N-terminally His_6_-tagged *hipA*	Laboratory collection
pET-15b	pBR322 *lacI* P_T7_, Amp^r^	Novagen
pKG127	pUC57::*hipBST_O127_*	Genscript
pSVN1	pBAD33::*hipT_O127_*, start codon GTG	This work
pSVN42	pEG25::*hipT_O127_*_::His6_, optimized SD, HipT_O127_ purification plasmid C-terminally His_6_-tagged *hipT_O127_*	This work
pSVN46	pEG25::*trpS*_His6_, optimized SD, TrpS purification plasmid harboring C-terminally His_6_-tagged *trpS*	This work
pSVN49	pEG25::*trpS^S197D^*_His6_, optimized SD, TrpS^S197D^ purification plasmid harboring C-terminally His_6_-tagged *trpS^S197D^*	This work
pSVN52	pEG25::*trpS^S197A^*_His6_, optimized SD, TrpS^S197A^ purification plasmid harboring C-terminally His_6_-tagged *trpS^S197A^*	This work
pSVN60	pUC57::_His6_*_-TEV_hipB*::*hipS*::*hipT_O127_*, optimized SDs for all genes	Genscript
pSVN61	pUC57::*hipB*::*hipS*::*hipT_O127_*_::His6_, optimized SDs for all genes used for TA complex purification via C-terminally His_6_-tagged *hipT_O127_*	Genscript
pSVN37	pEG25::*trpS*, optimized SD	This work
pSVN44	pBAD33::*hipBS_O127_*, optimized SD for *hipB_O127_*	This work
pSVN94	pET-15b::_His6_*_-TEV_hipB*::*hipS*::*hipT^D233Q^_O127_*, optimized SDs for all genes	This work
pSVN103	pNDM220::*trpS*, optimized SD	This work
pSVN109	pNDM220::*hipS_O127_*, optimized SD	This work
pSVN110	pNDM220::*hipBS_O127_*, optimized SDs	This work
pSVN111	pNDM220::*hipB_O127_*, optimized SD	This work
pSVN113	pUC57::*hipBST_Hi_*	Genscript
pSVN114	pUC57::*hipBST_Ta_*	Genscript
pSVN116	pNDM220::*hipT_O127_*, start codon GTG	This work
pSVN122	pNDM220::*hipB_Hi_*, optimized SD	This work
pSVN123	pNDM220::*hipS_Hi_*, optimized SD	This work
pSVN124	pNDM220::*hipBS_Hi_*, optimized SD for *hipB_Hi_*, overlapping *hipB_Hi_* and *hipS_Hi_*	This work
pSVN126	pNDM220::*hipB_Ta_*, optimized SD	This work
pSVN127	pNDM220::*hipS_Ta_*, optimized SD	This work
pSVN128	pNDM220::*hipBS_Ta_*, optimized SD for *hipB_Ta_*, overlapping *hipB_Ta_* and *hipS_Ta_*	This work
pSVN129	pBAD33::*hipT_Ta_*, optimized SD, start codon GTG	This work
pSVN135	pBAD33::*hipT_Hi_*, optimized SD	This work
pSVN138	pNDM220::*hipBS_Ta_*, optimized SDs	This work
pSVN139	pNDM220::*hipBS_Hi_*, optimized SDs	This work
pAH1	pNDM220::*hipA*	A. Harms
pAH2	pNDM220::*relE*	A. Harms

a SD, Shine-Dalgarno sequence.

**TABLE 3:** Oligonucleotides

Oligonucleotide	Sequence
FP1(GTG)	CCCCCGTCGACGGATCCAAGGAGTTTTATAAGTGGCGAATTGTCGTATTCTG
RP1	CCCCCGCATGCGAATTCGCTCACAGCAGCCCCAGACG
FP25	CCCCCTCGAGGGATCCAAAATAAGGAGGAAAAAAAAATGATCTGCTCAGGACCAC
RP15	GGGGGAATTCAAGCTTTCACTCGCCGATGCATAG
FP22	CCCCCTCGAGGGATCCAAAATAAGGAGGAAAAAAAAATGCATCGGCGAGTGAAAG
RP14	GGGGGAATTCAAGCTTTTATTCCTCCCAAGGTAAAATC
FP39	CCCCGGGGGATCCAAAATAAGGAGGAAAAAAAAATGAATTTTTGTCGTATTTTATTAAAG
RP21	GGGGGTACCCTGCAGTTATAGTTCAGGTTCATTTAATAG
FP29	CCCCCGGGGGATCCAAAATAAGGAGGAAAAAAAAATGGACAATCTTAGTGCAC
RP19	GGGGGTACCCTGCAGCTAAATCGCGCATAGTGAAAC
FP30	CCCCCGGGGGATCCAAAATAAGGAGGAAAAAAAAATGCGCGATTTAGTCCGC
RP20	GGGGGTACCCTGCAGTCATTGTTTTTCTTCCTG
FP42	AAAATAAGGAGGAAAAAAAAATGCGCGATTTAGTCCG
RP30	TTTTTTTTCCTCCTTATTTTCTAAATCGCGCATAGTGAAAC
FP34	CCCCCGGGGGATCCAAAATAAGGAGGAAAAAAAAGTGGACCGTTGTCTGATCAC
RP24	GGGGGTACCCTGCAGTTACCGGTCGAGATCGACAAC
FP32	CCCCCGGGGGATCCAAAATAAGGAGGAAAAAAAAATGAGCCATAGAAATCTACTCG
RP22	GGGGGTACCCTGCAGTTACTTTGCGGCCCATAACTTG
FP33	CCCCCGGGGGATCCAAAATAAGGAGGAAAAAAAAATGGGCCGCAAAGTAATTG
RP23	GGGGGTACCCTGCAGTTAATCATTAACCTCAAG
FP41	AAAATAAGGAGGAAAAAAAAATGGGCCGCAAAGTAATT
RP29	TTTTTTTTCCTCCTTATTTTCTATTTGGCGGCCCATAACTTGATAC
trpS Fw	CCCCCGGATCCAAAATAAGGAGGAAAAAAAAATGACTAAGCCCATCG
trpS RP4	GGGGGAATTCTTACGGCTTCGCCACAAAACC
trpS Rv	CCCCCAAGCTTTTACGGCTTCGCCACAAAAC
FP13	CCCCGGATCCAAAATAAGGAGGAAAAAAAAATGGCGAATTGTCGTATTC
RP5	GGGGAAGCTTTCAGTGATGGTGATGGTGATGCAGCAGCCCCAGACGATG
trpS RP3	GGGGGAAGCTTTTAGTGATGGTGATGGTGATGCGGCTTCGCCACAAAACC
trpS S197A Fw	AGAAGATGGCCAAGTCTGACGATAATCGC
trpS S197A Rv	AGACTTGGCCATCTTCTTGGTCGGCTC
trpS S197D Fw	AGAAGATGGACAAGTCTGACGATAATCGCA
trpS S197D Rv	CAGACTTGTCCATCTTCTTGGTCGGCTC
FP5	CCCCCGTCGACGGATCCAAGGAAAAAAAAAGTGGCGAATTGTCGTATTCTG
FP15	CCCCGTCGACAAAATAAGGAGGAAAAAAAAATGATCTGCTCAGGACCA
RP6	GGGGGCATGCTTATTCCTCCCAAGGTAAAA
hipX D233Q Fw	CGGTGTATCAGTTTGTTTCTGTCGCTCCC
hipX D233Q Rv	GAAACAAACTGATACACCGGCGCTAACG
FP16	CCCCGAATTCAAAATAAGGAGGAAAAAAAAATGCATCACCATCACCATCACGAAAACCTGTACTTCCAAGGGATCTGCTCAGGACCACAAAATC
RP7	GGGGAAGCTTTCACTCGCCGATGCATAGTTTC
RP13	GGGGGAATTCAAGCTTTTAGTGATGGTGATGGTGATGTTCCTCCCAAGGTAAAATC

### Gene knockout by P1 transduction.

To construct strain E. coli MG1655*ΔydeA*, gene knockout was obtained by phage P1 transduction using a strain of the Keio collection as donor according to standard procedure (46, 47).

### Multicopy suppression screening.

Genomic DNA (gDNA) of E. coli MG1655*ΔydeA* was purified according to the manufacturer’s instructions (EdgeBio). The gDNA was then partially digested with Sau3AI (Bsp143I) and fragments inserted into pBR322, which had been digested with BamHI and dephosphorylated. The gene library was transformed into a strain harboring the pBAD33::*hipT_O127_* plasmid and plated on agar plates containing arabinose.

### Site-directed mutagenesis.

Amino acid changes TrpS^S197A^, TrpS^S197D^, and HipT_O127_^D233Q^ were constructed by PCR mutagenesis (Table 3). The PCR products were digested with DpnI, and the resulting plasmids were transformed into E. coli strain DH5α.

### Protein purification.

HipT_O127_ (produced by pSVN42) was purified from E. coli strain BL21 that also produced HipB_O127_ and HipS_O127_ (pSVN44). Overnight cultures were diluted 100-fold into 350 ml fresh LB medium. At an optical density at 600 nm (OD_600_) of ≈0.3, the toxin gene was induced by the addition of 1 mM IPTG for 4 h, and cells were harvested by centrifugation. Pellets were resuspended in 25 ml cold buffer A (50 mM NaH_2_PO_4_ [pH 8], 0.3 M NaCl, 10 mM imidazole, 5 mM β-mercaptoethanol [BME]) with the addition of half a protease inhibitor cocktail each. Cells were carefully sonicated for 5 min at 60% amplification (2 s on and 2 s off) while still kept cold. The cell lysate was spun at 16,000 rpm for 30 min at 4°C, and the cleared lysate was incubated at 4°C for 1 h with 1 ml Ni beads that had been freshly equilibrated in the same buffer for 1 h. Protein-bound beads were then applied to gravity flow columns and washed with 50 ml of buffer B (50 mM NaH_2_PO_4_ [pH 8], 0.3 M NaCl, 35 mM imidazole, 1 mM BME). As described previously, the toxin and antitoxins were separated with urea washes to leave the His-tagged protein on the affinity column (48). His-tagged proteins were purified according to the manufacturer’s protocol, further purified using an Äkta Pure (GE Healthcare) fast protein liquid chromatography (FPLC) instrument, and stored in 200 mM NaCl, 50 mM Tris-HCl, and 5% glycerol. All proteins purified with His tags were tested and compared to wild-type proteins *in vivo* prior to purification in order to assess their functionality.

### Phosphorylation *in vitro*.

Phosphorylation reactions were performed in the presence of 0.05 μl [γ-^32^P]ATP (6,000 Ci/mmol; Perkin Elmer) per microliter of reaction mixture, 66.6 μM ATP (nonradioactive), and aminoacylation buffer (1 mM dithiothreitol [DTT], 10 mM KCl, 16 μM ZnSO_4_, and 20 mM MgCl_2_) for 45 min as described previously (11). Each reaction was stopped by the addition of 1 volume Laemmli loading buffer, the reaction mixture was incubated for 10 min at 95°C, and proteins were resolved by SDS-PAGE and exposed using phosphorimaging (GE Healthcare) overnight.

### Phosphorylation *in vitro* measured by LC-MS.

The phosphorylation reaction was performed with 13.5 μM TrpS and 6.7 μM HipT_O127_ in the presence of 5 mM ATP and aminoacylation buffer for 45 min at 37°C. The reaction was stopped by the addition of 4 volumes of denaturation buffer (6 M urea, 2 M thiourea, 1 mM DTT, and 10 mM Tris-HCl, pH 8.0), and the reaction mixture incubated for 30 min at room temperature, followed by incubation with 5.5 mM iodoacetamide for 45 min at room temperature. Denatured proteins were digested overnight either with endoproteinase Lys-C (1:100 [wt/wt]; Wako) in 20 mM bicarbonate, pH 8.0, or with endoproteinase Arg-C (1:100 [wt/wt]; Roche) in 90 mM Tris-HCl, pH 7.6, 8.5 mM CaCl_2_, 5 mM DTT, 0.5 mM EDTA. Digested peptides were purified via Pierce C18 Spin Tips, and 0.5 μg of each sample was measured by liquid chromatography-tandem mass spectrometry (LC-MS/MS) as described previously (49). Briefly, peptides were separated by an Easy-nL 1200 ultra-high-performance liquid chromatography (UHPLC) instrument (Thermo Fisher Scientific) and transferred into an online coupled Q Exactive HF mass spectrometer (Thermo Fisher Scientific) by nanoelectrospray ionization. Peptides were eluted from a 20-cm-long analytical column packed with 1.9-μm reverse-phase particles using a 33-min segmented gradient of 5% to 50% solvent B (80% [vol/vol] acetonitrile, 0.1% [vol/vol] formic acid) at a constant flow rate of 300 nl/min. Full-scan MS spectra were acquired in a mass range from 300 to 1,650 *m/z* with a maximum injection time of 45 ms and a resolution of 60,000. Higher-energy collisional dissociation MS/MS scans of the 7 (Top7 data-dependent method) most abundant peaks were recorded with a maximum injection time of 220 ms at a resolution of 60,000. Acquired raw data were processed with MaxQuant software (version 1.5.2.8) (50) using default settings if not stated otherwise. The derived peak list was searched against a reference E. coli K-12 proteome (Taxon identifier 83333) containing 4,324 entries (UniProt, release 2017/12), the protein sequence of HipT, HipS, and HipB from E. coli O127:H6, and a file containing 245 common laboratory contaminants using a built-in Andromeda search engine (51). Methionine oxidation, protein N-terminal acetylation, and Ser/Thr/Tyr phosphorylation were defined as variable modifications, and carbamidomethylation of cysteines was set as a fixed modification. The maximum number of missed cleavages allowed was set to 3 for the endoproteinase Lys-C and to 2 for Arg-C. Only phosphopeptides with an Andromeda score of >70 and a localization probability of >0.75 were considered, and their MS/MS spectra were inspected manually (Fig. S10).

10.1128/mBio.01138-19.10 FIG S10 Representative mass spectra after *in vitro* kinase assay revealing HipT_O127_-mediated phosphorylation sites on TrpS (A, B) and autophosphorylation sites of HipT_O127_ (C, D). Download FIG S10, PDF file, 0.2 MB. Copyright © 2019 Nielsen et al. 2019 Nielsen et al. This content is distributed under the terms of the Creative Commons Attribution 4.0 International license.

### Measurement of cellular (p)ppGpp levels.

Measurement of cellular (p)ppGpp levels was performed as described previously (52, 53).

### Mass spectrometry.

The mass spectrometry proteomics data have been deposited to the ProteomeXchange Consortium via the PRIDE (54) partner repository with the data set identifier PXD012023.

### Construction of plasmids.

Construction of plasmids is summarized below.

### pKG127.

The region of the E. coli O127 E2348/69 genome (accession number NC_011601.1) containing the *hipBST* locus (+3,948,403 to +3,950,320) was synthesized (GeneScript) and inserted into the SalI restriction site of pUC57.

### pSVN1.

*hipT_O127_* with start codon GTG was amplified from pKG127 using primers FP1(GTG) and RP1. The resulting PCR product was digested with SalI and SphI and ligated with pBAD33.

### pSVN37.

*trpS* was amplified from pCA24N::*trpS* from the ASKA collection (55) using primers trpS Fw and trpS Rv. The resulting PCR product was digested with BamHI and HindIII and ligated into pEG25.

### pSVN42.

*hipT_O127_*_::His6_ was amplified from pKG127 using primers FP13 and RP5. The resulting PCR product was digested with BamHI and HindIII and ligated into pEG25.

### pSVN44.

*hipBS_O127_* was amplified from pKG127 using primers FP15 and RP6. The resulting PCR product was digested with SalI and SphI and ligated into pBAD33.

### pSVN46.

*trpS*_His6_ was amplified from pSVN37using primers trpS Fw and trpS RP3. The resulting PCR product was digested with BamHI and HindIII and ligated into pEG25.

### pSVN49.

The mutation in *trpS^S197D^*_His6_ was created using pSVN46 and primers trpS S197D Fw and trpS S197D Rv in a site-directed plasmid mutagenesis PCR. The fragment was digested with DpnI before being transformed into E. coli DH5α.

### pSVN52.

The mutation in *trpS^S197A^*_His6_ was created using pSVN46 and primers trpS S197A Fw and trpS S197A Rv in a site-directed plasmid mutagenesis PCR. The fragment was digested with DpnI before being transformed into E. coli DH5α.

### pSVN94.

_His6_*_-tev_hipB_O127_*::*hipS_O127_*::*hipT_O127_* with optimized SDs for all three genes was subcloned from pSVN60 by digesting with XbaI and XhoI, purifying the DNA fragment from a 1% agarose gel, and ligating into pET-15b. The mutation in _His6_*_-tev_hipB_O127_*::*hipS*::*hipT^D233Q^* was created using primers hipX D233Q Fw and hipX D233Q Rv in a site-directed plasmid mutagenesis PCR. The fragment was digested with DpnI before transformation.

### pSVN103.

*trpS* was amplified from pCA24N::*trpS* from the ASKA collection (55) using primers trpS Fw and trpS RP4. The resulting PCR product was digested with BamHI and EcoRI and ligated into pNDM220.

### pSVN109.

*hipS_O127_* was amplified from pKG127 using primers FP22 and RP14. The resulting PCR product was digested with XhoI and EcoRI and ligated into pNDM220.

### pSVN110.

*hipBS_O127_* was amplified from pSVN61 using primers FP25 and RP14. The resulting PCR product was digested with XhoI and EcoRI and ligated into pNDM220.

### pSVN111.

*hipB_O127_* was amplified from pSVN61 using primers FP25 and RP15. The resulting PCR product was digested with XhoI and EcoRI and ligated into pNDM220.

### pSVN113.

The region of the H. influenzae Rd KW20 genome (NC_000907.1) containing the *hipBST* locus (+710,585 to +712,589) was synthesized and inserted into the SalI site of pUC57 (GeneScript).

### pSVN114.

The region of the Tolumonas auensis DSM 9187 genome (NC_012691.1) containing the *hipBST* locus (+2,117,168 to +2,119,170) was synthesized and inserted into the SalI site of pUC57 (GeneScript).

### pSVN116.

*hipT_O127_* was amplified using primers FP5 and RP1 from pSVN1. The fragment was then cloned into cut pNDM220 using BamHI and EcoRI, resulting in pSVN116 (pNDM220::*hipT*_O127_).

### pSVN122.

*hipB_Hi_* was amplified from pSVN113 using primers FP29 and RP19. The resulting PCR product was digested with BamHI and KpnI and ligated into pNDM220.

### pSVN123.

*hipS_Hi_* was amplified from pSVN113 using primers FP30 and RP20. The resulting PCR product was digested with BamHI and KpnI and ligated into pNDM220.

### pSVN124.

*hipBS_Hi_* was amplified from pSVN113 using primers FP29 and RP20. The resulting PCR product was digested with BamHI and KpnI and ligated into pNDM220.

### pSVN126.

*hipB_Ta_* was amplified from pSVN114 using primers FP32 and RP22. The resulting PCR product was digested with BamHI and KpnI and ligated into pNDM220.

### pSVN127.

*hipS_Ta_* was amplified from pSVN114 using primers FP33 and RP23. The resulting PCR product was digested with BamHI and KpnI and ligated into pNDM220.

### pSVN128.

*hipBS_Ta_* was amplified from pSVN114 using primers FP32 and RP23. The resulting PCR product was digested with BamHI and KpnI and ligated into pNDM220.

### pSVN129.

*hipT_Ta_* was amplified from pSVN114 using primers FP34 and RP24. The resulting PCR product was digested with XmaI and PstI and ligated into pBAD33.

### pSVN135.

*hipT_Hi_* was amplified from pSVN113 using primers FP39 and RP21. The resulting PCR product was digested with XmaI and PstI and ligated into pBAD33.

### pSVN138.

The optimized SD inserted between *hipB_Ta_* and *hipS_Ta_* was created using pSVN128 and primers FP41 and RP29 in a site-directed plasmid mutagenesis PCR. Eight reactions were carried out at different temperatures with a gradient PCR. The samples were pooled and digested with DpnI to digest the parental plasmid before being transformed into E. coli DH5α.

### pSVN139.

The optimized SD inserted between *hipB_Hi_* and *hipS_Hi_* was created using pSVN124 and primers FP42 and RP30 in a site-directed plasmid mutagenesis PCR. The fragment was digested with DpnI before transformation.
