# Supplementary material for: Serine-Threonine Kinases Encoded by Split hipA Homologs Inhibit Tryptophanyl-tRNA Synthetase
Source: mBio. 2019 Jun 18;10(3):e01138-19. doi: 10.1128/mBio.01138-19 (PMC6581861; doi:10.1128/mBio.01138-19)
Supplement: FIG S1 [file mBio.01138-19-sf001.pdf]

A

```
                        74                                                                      146
HipA E. coli K-12       RIVKRYHAKSRQPFDLLSEIGRDSVGAVTLIPEDETVTHPIMAWEKLTEARLEEVLTAYKADIPLGMIREEND
HipT E. coli O127       ---------MANCRILLTPLNERDEQRG-YSTQGLKRLSGTAKLNPRLGFTRTQFVQELPRQQ----------
HipT H. infl KW20       ---------MNFCRILLKPLKKNEALSG-YSAEGLHYLTDNKHFNPELPFSRQEFITVKPQKQ----------
HipT T. auensis         ---------MDRCLITLELLDKKQPQPG-YSSRGIKYLTGNITTSMELPFTRDEFITELPGLQ----------
HipT V. cholera O1       -----MDLSNKSLISLDELNDQNREQGEYKKGEIKTLLGSNRFQMQLPFTRGDFVKEMPKRQ----------
HipT V. halioticoli      -----MYPSDRSLISLALLDSNTRYQDEYLRSEVKELLGSSRNKMQLPFTREDFLIEGPIKQ----------
                        147                                                                     219
HipA E. coli K-12       FRISVAGAQEKTALLRIGNDWCIPKGITPTTHIIKLPIGEIRQPNATLDLSQSVDNEYYCLLLAKELGLNVPD
HipT E. coli O127       KGMSISGYQPKLQLVLDEG----------EFRVVDHQGNFILKP-SPADFPGLAENEHATMTLMSRLGFDVPV
HipT H. infl KW20       QGMSISGFQPKLQLIIKDE----------HFDSVNQQGNYILKP-SPEEYPFLAENEHATMRIMKELGFDVPE
HipT T. auensis         QGLSISGYQPKLSLAIQNK----------QFHVVDQMGSYILKP-SPEAYPYLAENEHATMMVMKKLKFSVPE
HipT V. cholerae O1     KGMSISGYQPKLSLAINEHG---------QLSVVTNKALFILKP-SPEEFPHLAENEHATMLVMKMLGFDTPP
HipT V. halioticoli     KGMSISGYQPKLSLALDADG---------LLNMVEHQAQFILKP-SPQNFPNLAANEHATMLAMKQLGFDLPP
```

Autophosphorylation↑ P-Loop — Activation Loop

```
                        220                                                                     292
HipA E. coli K-12       AEIIKAGNVR-------ALAVERFDRRWNAERTVLLRLPQEDMCQTFGLPSSVKYESDGGPGIARIMAFLMGS
HipT E. coli O127       HGLLSFAPQS-EEELEYAFVIRRYDRDNKG--LPVHQEQLDGAMQITDKYGKTGNDNEQYVSYETLARFLVAH
HipT H. infl KW20       NGLVSFAGE--QNHKEFAFVITRFDRDKQQ--KPMHQEQLDGAMNIRDKYGKIGADNEQYVSYEQIAKFILQH
HipT T. auensis         FGLVAFKKVDNADPAEFAFIIKRFDRDMKTG-EKIHQEQLDGAMAIDDKYGHR--DGKQQISYESACRFLIEQ
HipT V. cholerae O1     FGLVRFSDPN--NLGELAFLIRRYDRLNAGE-EAIHQEQLDAAMGVPEKYGKLKDDNEGYVSYEQACKFLLEN
HipT V. halioticoli     FGLVRFKSEL--SDGEVAFLIRRYDRRLFDK-EVIHQEQLDGAMGVAEKYGKIKNDDEGYISYEQACLFMIRN
                        293                                                                     365
HipA E. coli K-12       SE-ALKDRYDFMKFQVFQWLIGATDGHAKNFSVFIQAGGSYRLTPFYDIISAFPVLGGTGIHISDLKLAMGLN
HipT E. coli O127       VNDNIAFKIDLFRRIVYAWLLGNNDMHLRNFGLVYSDGLTPALAPVYDFVSVAPYPEYFYS-NYLALPLLTRE
HipT H. infl KW20       TENHLAQQREIFRRIIYAYLLGNNDLHLRNFSFIYPKNSHPKLAPIYDFVSVSPYPEIFNS-TLLALPLLARE
HipT T. auensis         VDSSLPFKRDLFLRVIYAYLLGNNDFHLRNFGILEPQVAPNLLAPVYDFVSTAPYSSTFNS-CYLALPLLTRE
HipT V. cholerae O1     VESSLTFKMELFNRVLTAYFLGNNDLHLRNIGILLPEHEAPKIAPIYDYVSIAPYSEYIANESSLALPLLASE
HipT V. halioticoli     IDSSLAFKRELFNRVLAAYFLGNNDLHLRNFGLLHPLGQKATLAPVYDYVSVAPYKGYIANENSLALPLLAEE
```

Conserved catalytic motif — Mg2+ binding

```
                        366                                                                     438
HipA E. coli K-12       AS-----KGKKTAIDKIYPRHFLATAKVLRFPE-VQMHEILSDFARMIPAALDNVKTSLPTDFPENVVTAVES
HipT E. coli O127       EGGRELAPGFHSDYGEYIGQDFLLLGESMGLAP-RLLEKLFQDIRKENAIVMETYEQSFMTQDHIQAVLQCYR
HipT H. infl KW20       EGNATLAKGFNTQYGEYIGDDFVEFGENIGLNKNVIIQKLIPEIIQEKEKVEQIYSQSFMPQPHIDCVLKTYR
HipT T. auensis         ENDRALAPGFDTQFGQYIGYDFIAFGKGIGLNE-RYILKLFAEIQKQADVVRDTYQISFMPPQHIEAVLKCFN
HipT V. cholerae O1     EGNNRNTSGC-LSHCTYTGHDFITFGECIGLNP-KLAIKLIKGLLAKKDQILGIYRNSFMPDYQILKVEEWIE
HipT V. halioticoli     EGNNNASSGQ-HSHCTYTGYDFLNFGEKIGLSR-KLAQKLITTLLKQKNKIKCIYQDSLMPKADVDKVLEWID
                        439          454
HipA E. coli K-12       NVLRLHGRLSREYGSK
HipT E. coli O127       HRLGLL----------
HipT H. infl KW20       KRLALLNLLNEPEL--
HipT T. auensis         TRLKLIEVVDLDR---
HipT V. cholerae O1     SRIHYLNVLDPVKT--
HipT V. halioticoli     SRERYLQVNQHVDI—
```

B

```
                        1                                                                       73
HipA E. coli K-12       ---MPKLVTWMNNQRVGELTKLANGAHTFKYAPEWLASRYARPLSLSLPLQRGNITSDAVFNFFDNLLPDSPI
HipS E. coli O127       ---MHRRVKVLLYGQVVGELSQNDSGFLFQYAHDYHG----PAISISLPVAQRQFPSETLHPYFASLAPEG-W
HipS H. infl KW20       MRDLVRSGKVFLYGEFIGLLREDHRGFHFSYNPDYQG----IPLSLSFPIEQSPFHSDTLFPYFASLVPEG-W
HipS T. auensis         ---MGRKVIVFCYGKQAGILSENNDEYVFTYDDEYRG----KPISLSLPLSKKTFPSKGLHPFFKTLAPEG-W
HipS V. cholerae O1     ---MDRKVKVLLYGKFCGILSQDDRGFTFEYDQSYKG----RSLSLSMPITVGTFTSSELHPFFQSLAPEG-W
HipS V. halioticoli     ---MDRKVKVLLYGEFCGVLTQSATGYVFEYDKNYRG----QSLSLSLPVSTRRFETEQLHPYFQSLAPEG-W
                        74                                       115
HipA E. coli K-12       VRDRIVKRYHAKSRQPFDLLSEIGRDSVGAVTLIPEDETVTH
HipS E. coli O127       LRQRYSQIQHRDENDLLGMLIDNGKNLLGAIQILPWEE----
HipS H. infl KW20       LKHKYALHQRIDESDMFRFLLNNGENMLGAVQIQEEKQ----
HipS T. auensis         LKKRYSELQHIDERDLFGFLIRNGSDLLGAITFLEVND----
HipS V. cholerae O1     LKKRYSELQKIDENDPLGMLLSNGQNLLGAVQLLRLDDDGSE
HipS V. halioticoli     LKRSFSEQQKIDERDILGMLIANGYDLLGAVQLMRLDDVS—
```

C

```
                        1                                                                       73
HipB E. coli O127       MICSGPQNLAQELKAIGDQLQELQKKLIQGPNIS--QPRPLLTIETPRHLGEQLNARRKELGIDLYTLELQTG
HipB H. infl KW20       -----MDNLSAQLENQIDELKKLQKSVITYEQAQ-RQQLYAGKITDLKAFGKMLNDKRKSLGIELSMLELQTG
HipB T. auensis         MSHRNLLELRKQSDELFQH----FSRLLDNAEKELTEPELGIAADNYDAMTLMLKTKRKSLGLTLSDLELQTG
HipB V. cholerae O1     -MTPKVLQLRELANKVEANLHSALEEMLHQVEISSQRPITSYSMYDLDTLTQDMREKKK--MITLEDLELQTG
HipB V. halioticoli     -MNPKLFQLRKELQHISAN----MEEILSDIEDASNSPVPSYSIFDKKLLINDLRERRK--GISYEDLELQTD
```

HTH Motif

```
                        74                                                                    143
HipB E. coli O127       ISTSTLKRLFKDPEQVKFGSVFAVANVLGVKLCIGE-----------------------------------
HipB H. infl KW20       VSISTLNRLFQDPSQVRFTTVFLVAQTLGVSLCAI------------------------------------
HipB T. auensis         ISQSTLKRLFSDPENARVGHLLLVCQELGIKLWAAK-----------------------------------
HipB V. cholerae O1     ISSSTIKRMLKNPRNTSLENFLDVANELGMKIWIEK-----------------------------------
HipB V. halioticoli     ISRSTLMRIMKDPANTSLENFLVVANELGMKIWDRKVKVLLYGEFCGVLTQSATGYVFEYDKNYRGQIEK
```

HTH Motif

Figure S1 - page 1

D

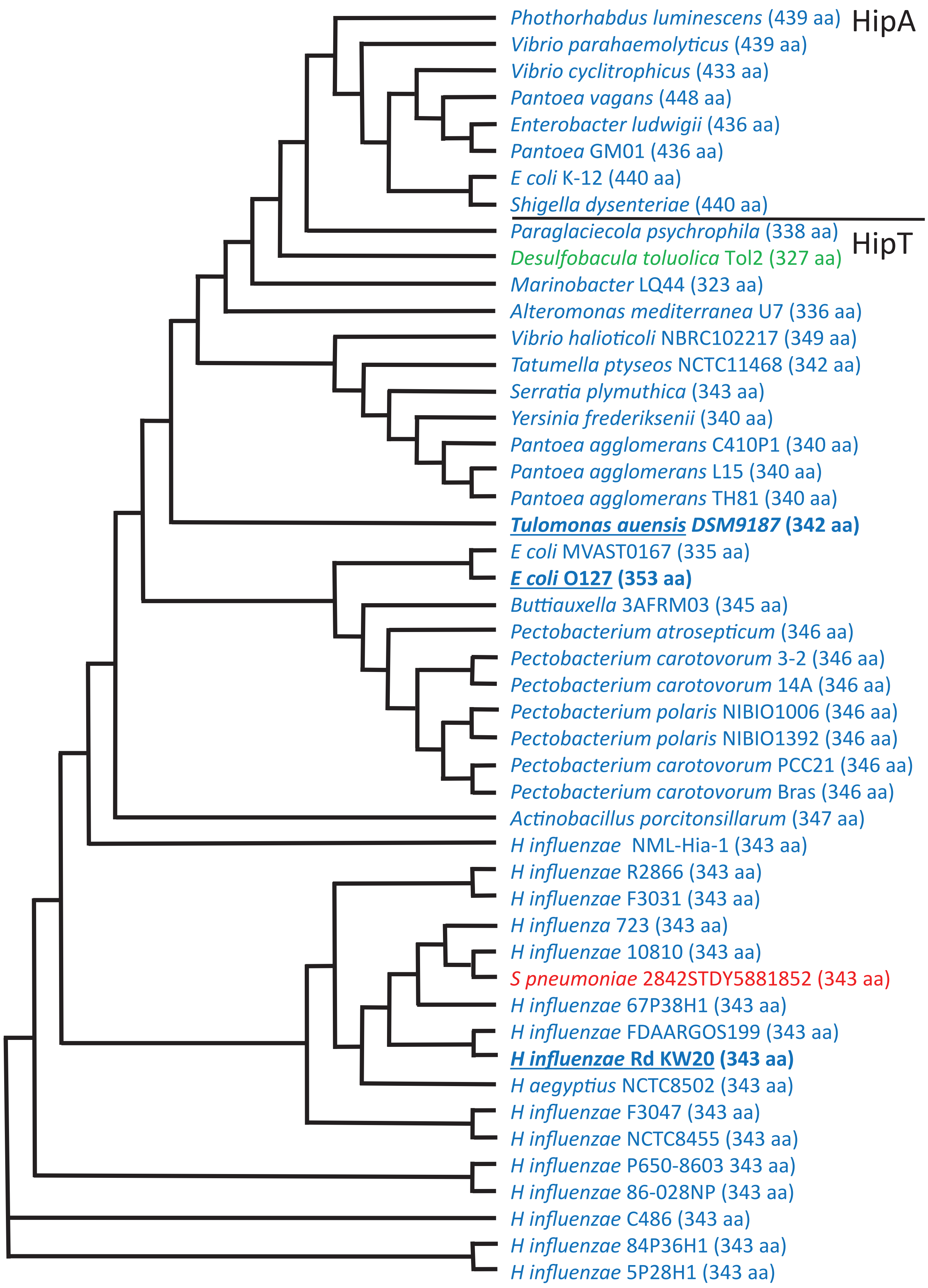

Figure S1 - page 2

Figure S1. Alignments of protein sequences encoded by *hipBST* loci from five different gammaproteobacteria and a phylogenetic analysis of HipT

(**A**) Alignment of HipT homologs with the C-terminal part of HipA of *E. coli* K-12. The eight aa conserved P-loop (34) in HipA indicated below the sequences was used as a fix point to generate the alignment. The auto-phosphorylated Ser150 in HipA is fully conserved in the HipT homologs and the HipT proteins contain conserved catalytic domains and Mg^{2+} binding motifs as indicated (34, 36, 62). The HipT sequences were from *E. coli* O127:H6 strain E2348/69 (GenBank: CAS11333.1), *H. influenza* Rd KW20 (NCBI: NP_438824), *Tolumonas auensis* DSM 9187 (NCBI: WP_015879003.1), *Vibrio cholera* O1 strain NHCM-017 (WP_050916634.1) and *V. halioticoli* (GenBank: GAD88429.1).

(**B**) Alignment of five HipS homologs encoded by genes just upstream of the genes encoding the HipT homologs shown in (A) with the N-terminal part of HipA. The HipS sequences were from *E. coli* O127:H6 strain E2348/69 (GenBank: CAS11334.1), *H. influenza* Rd KW20 (NCBI: NP_438825.1), *Tolumonas auensis* (NCBI: WP_015879002.1), *V. cholorae* O1 strain NHCM-017 (WP_044125702.1) and *Vibrio halioticoli* (GenBank: GAD88428.1).

(**C**) Alignment of five HipB homologs encoded by genes just upstream of the genes encoding the HipS homologs shown in (B). The HipB sequences were from *E. coli* O127:H6 strain E2348/69 (GenBank: CAS11335.1), *H. influenza* Rd KW20 (NCBI: NP_438826.1), *Tolumonas auensis* (NCBI: WP_083757795.1), *V. cholerae* O1 strain NHCM-017 (WP_050916568.1) and *V. halioticoli* (GenBank: AD88427.1). Color-codes of the alignments: black on white: non-similar residues; blue on cyan: consensus residue derived from a block of similar residues at a given position; black on green: consensus residue derived from the occurrence of greater than 50% of a single, distinct residue; red on yellow: consensus residue derived from a completely conserved residue; green on white: residue weakly similar to consensus residue. The alignments in Figs S1A, B and C were generated by the commercial version Vector NTI (Invitrogen).

(**D**) Phylogenetic tree of 8 HipA and 40 HipT homologs. A sequence alignment of the proteins were accomplished using an online version of MUSCLE (63) provided by European Bioinformatics Institute at EMBL and used to derive the phylogenetic tree presented as a cladogram. Sampling of the HipA and HipT sequences was accomplished using BLASTP at NCBI and was not exhaustive. Accession numbers of the proteins included in the cladogram were as follows: *P. luminescens* TT01: CAE17272.1; *V. parahaemolyticus*: WP_020904255.1; *V. cyclitrophicus*: WP_102385597.1; *Pantoea vagans*: WP_095707190.1; *Enterobacter ludwigii*: WP_081112566.1; *Pantoea* GM01: EJL89497.1; *E. coli* K-12: P23874.2; *S. dysenteriae*: WP_119170656.1; *Paraglaciecola psychrophila* 170: AGH46782.1; *Desulfobacula toluolica* Tol2: CCK78946.1; *Marinobacter* LQ44: AMQ90498.1; *Alteromonas mediterranea* U7: AGP89851.1; *V. halioticoli* NBRC 102217: GAD88429.1; *Tatumella ptyseos* NCTC11468: SQK77238.1; *S. plymuthica* PRI-2C: ANS44223.1; *Y. frederiksenii* FDAARGOS 417: ATM86408.1; *Pantoea agglomerans* C410P1: AOE38443.1; *Pantoea agglomerans* L15: AZI51552.1; *Pantoea agglomerans* TH81: AYP22744.1; *T. auensis* DSM 9187: ACQ93535.1; *E. coli* MVAST0167: AML11457.1; *E. coli* O127:H6 E2348/69: CAS11333.1; *Buttiauxella* 3AFRM03: AYN26082.1; *P. atrosepticum* 36A: ATY88941.1; *P. carotovorum* 3-2: AVT56767.1; *P. carotovorum* 14A: AZK60881.1; *P. Polaris* NIBIO1006: ASY75229.1; *P. Polaris* NIBIO1392: ASY81571.1; *P. carotovorum* PCC21: AFR01461.1; *P. carotovorum* subsp Bras: ARA78327.1; *A. porcitonsillarum* 9953L55: AWI50429.1; *H. influenzae* NML-Hia-1: AOZ67195.1; *H. influenzae* R2866: ADO81720.1; *H. influenzae* F3031: CBY81777.1; H. influenza 723: AJO91476.1; *H. influenzae* 10810: CBW28981.1; *S. pneumonia* 2842STDY5881852: CVQ15586.1; *H. influenzae* 67P38H1: AVJ03036.1; *H. influenzae* FDAARGOS199: ARB90233.1; *H. influenzae* Rd KW20: NP_438824; *H. aegyptius* NCTC8502: SQH35811.1; *H. influenzae* F3047: CBY86290.1; *H. influenzae* NCTC8455 SQK92775.1; *H. influenzae* P650-8603: AXP41025.1; *H. influenzae* 86-028NP: AAX87695.1; *H. influenzae* C486: AJO89092.1; *H. influenzae* 84P36H1: AWP55634.1; *H. influenzae* 5P28H1: AVI99364.1.
