# Supplementary material for: Serine-Threonine Kinases Encoded by Split hipA Homologs Inhibit Tryptophanyl-tRNA Synthetase
Source: mBio. 2019 Jun 18;10(3):e01138-19. doi: 10.1128/mBio.01138-19 (PMC6581861; doi:10.1128/mBio.01138-19)
Supplement: FIG S2 [file mBio.01138-19-sf002.pdf]

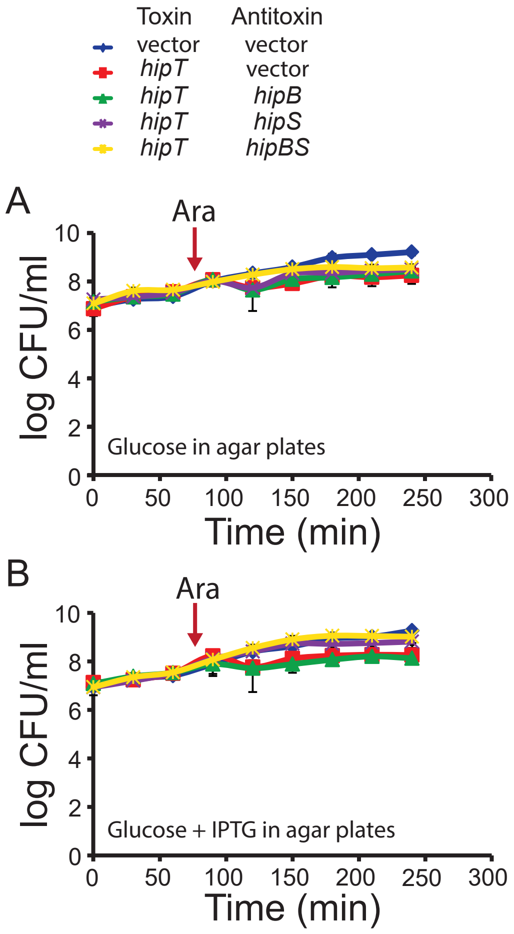

Figure S2. Long-term incubation shows HipT of *E. coli* O127 induces bacteriostasis. Viable counts (CFU/ml) before and after *hipT*$_{O127}$ induction in the presence or absence of induction of *hipB*$_{O127}$ or *hipS*$_{O127}$ and *hipBS*$_{O127}$. Cultures of MG1655 harboring pSVN1 (pBAD33::*hipT*$_{O127}$) or the empty pBAD33 vector combined with pSVN111 (pNDM220::*hipB*$_{O127}$), pSVN109 (pNDM220::*hipS*$_{O127}$), pSVN110 (pNDM220::*hipBS*$_{O127}$) or the empty low-copy-number pNDM220 vector growing exponentially in LB medium at 37°C. The pBAD promoter was induced by the addition of arabinose (0.2%) at OD_{600} ≈0.3 (red arrow). At each time point, 0.5 ml cell samples were washed in PBS before 10 times dilution series were spotted on agar plates with (**A**) glucose only (0.2%), to repress *hipT*$_{O127}$ expression or (**B**) glucose plus µM IPTG (200 µM) to also induce *hipB*$_{O127}$ or *hipS*$_{O127}$ and *hipBS*$_{O127}$ on the plates. Plates were incubated for 40 hours at 37°C before counting. Data points represent means of results from at least three independent experiments, and error bars indicate standard deviations. As seen, the prolonged incubation period (16h versus 40h) allowed for the full recovery of viability, even in the absence of *hipS* or *hipBS* (compare Fig. S2 with Figs 1C, D).
