# Supplementary material for: Serine-Threonine Kinases Encoded by Split hipA Homologs Inhibit Tryptophanyl-tRNA Synthetase
Source: mBio. 2019 Jun 18;10(3):e01138-19. doi: 10.1128/mBio.01138-19 (PMC6581861; doi:10.1128/mBio.01138-19)
Supplement: FIG S3 [file mBio.01138-19-sf003.pdf]

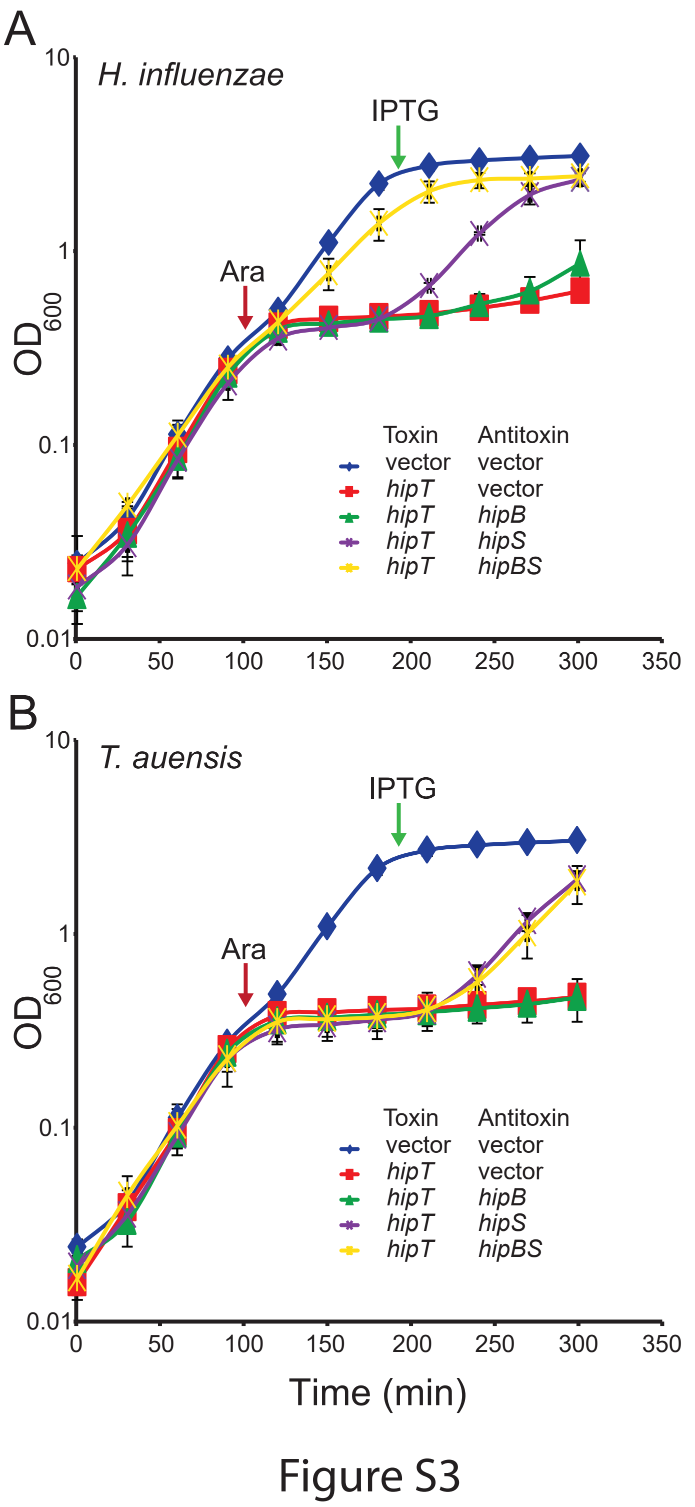

Figure S3. HipT of *H. influenzae* and *T. auensis* inhibit cell growth and can be neutralized by cognate HipS.

Cells were grown as described in the Legend to Fig. 1C. As seen, cognate HipB_{Hi} augments HipS_{Hi} in the neutralization of HipT_{Hi}. Strains used in (**A**) were MG1655 harbouring pSVN135 (pBAD33::*hipT*$_{Hi}$) or the empty pBAD33 vector combined with pSVN122 (pNDM220::*hipB*$_{Hi}$), pSVN123 (pNDM220::*hipS*$_{Hi}$) or pSVN139 (pNDM220::*hipBS*$_{Hi}$) or the empty low-copy-number pNDM220 as indicated and strains in (**B**) were MG1655 harbouring pSVN129 (pBAD33::*hipT*$_{Ta}$) or the empty pBAD33 vector combined with pSVN126 (pNDM220::*hipB*$_{Ta}$), pSVN127 (pNDM220::*hipS*$_{Ta}$) or pSVN138 (pNDM220::*hipBS*$_{Ta}$) or the empty low-copy-number pNDM220 as indicated. Data points represent means of results from at least two independent experiments, and error bars indicate standard deviations.
