# Supplementary material for: Serine-Threonine Kinases Encoded by Split hipA Homologs Inhibit Tryptophanyl-tRNA Synthetase
Source: mBio. 2019 Jun 18;10(3):e01138-19. doi: 10.1128/mBio.01138-19 (PMC6581861; doi:10.1128/mBio.01138-19)
Supplement: FIG S4 [file mBio.01138-19-sf004.pdf]

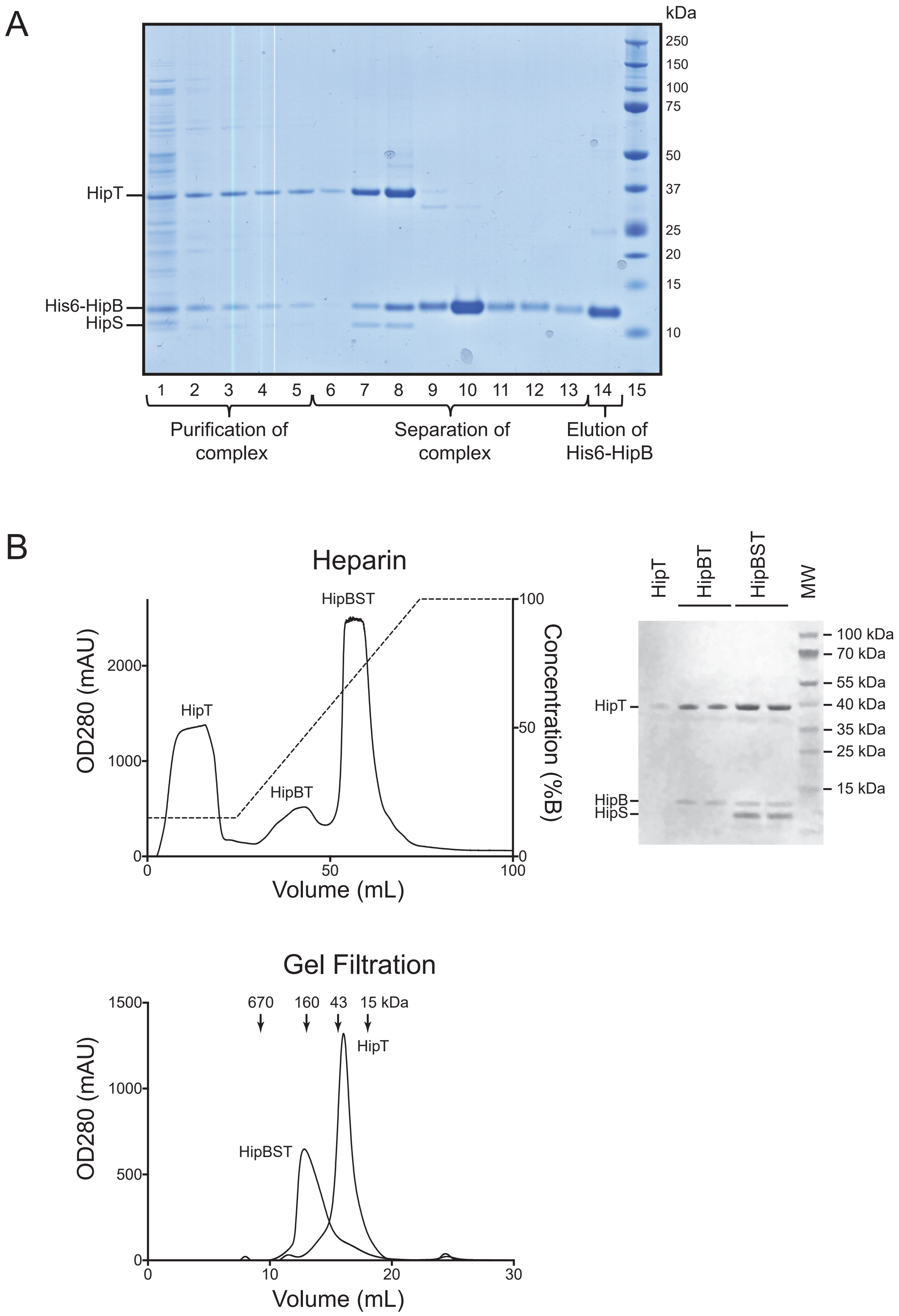
A
kDa
250
150
100
75
50
37
25
20
15
10
HipT
His6-HipB
HipS
1 2 3 4 5 6 7 8 9 10 11 12 13 14 15
Purification of complex
Separation of complex
Elution of His6-HipB
B
Heparin
HipBST
HipT
HipBT
OD280 (mAU)
Concentration (%B)
Volume (mL)
HipT
HipBT
HipBST
MW
100 kDa
70 kDa
55 kDa
40 kDa
35 kDa
25 kDa
15 kDa
HipB
HipS
Gel Filtration
670
160
43
15 kDa
HipT
HipBST
OD280 (mAU)
Volume (mL)

Figure S4

Figure S4. HipB$_{O127}$, HipS$_{O127}$ and HipT$_{O127}$ form a complex.
(**A**) Purification of His$_6$-TEV-HipB$_{O127}$, HipS$_{O127}$ and HipT$^{D233Q}_{O127}$ from strain BL21 containing pSVN94 analysed by SDS-PAGE. Strain BL21 containing pSVN94 was grown in LB medium at 37°C and His$_6$-TEV-HipB$_{O127}$ purified according to standard procedure. As seen, the N-terminally his-tagged HipB$_{O127}$ pulled down HipS$_{O127}$ and HipT$_{O127}$. Lanes 1-5 contained samples from washes with buffer B (50 mM NaH_2PO_4 (pH 8), 0.3 M $NaCl_2$, 35 mM imidazole and 1 mM β-mercaptoethanol). Lane 6-10 show samples from washes with buffer C (100 mM NaH_2PO_4 (pH 8), 10 mM Tris-HCl (pH 8) and 1 mM β-mercaptoethanol) with different concentrations of urea: 0 M, 2.45 M, 4.9 M, 7.35 M and 9.8 M urea, respectively. Lane 11 shows a sample from flow-through after overnight wash with buffer C containing 9.8 M urea. Lanes 12-13 show samples from additional washes with buffer C containing 9.8 M urea after overnight incubation. Lane 14 contain a sample from elution with buffer D (100 mM NaH_2PO_4 (pH 8), 10 mM Tris-HCl (pH 8), 9.8 M urea, 0.5 M imidazole and 1 mM β-mercaptoethanol). (**B**) Top; purification of HipBST$_{O127}$ subcomplexes using a Heparin column and elution using increasing concentrations of salt. Bottom; purification of HipBST$_{O127}$ and isolated HipT$_{O127}$ toxin on a gel filtration column. Elution positions of standard proteins with known mass (indicated in kDa) are shown with vertical arrows.
