# Supplementary material for: Serine-Threonine Kinases Encoded by Split hipA Homologs Inhibit Tryptophanyl-tRNA Synthetase
Source: mBio. 2019 Jun 18;10(3):e01138-19. doi: 10.1128/mBio.01138-19 (PMC6581861; doi:10.1128/mBio.01138-19)
Supplement: FIG S5 [file mBio.01138-19-sf005.pdf]

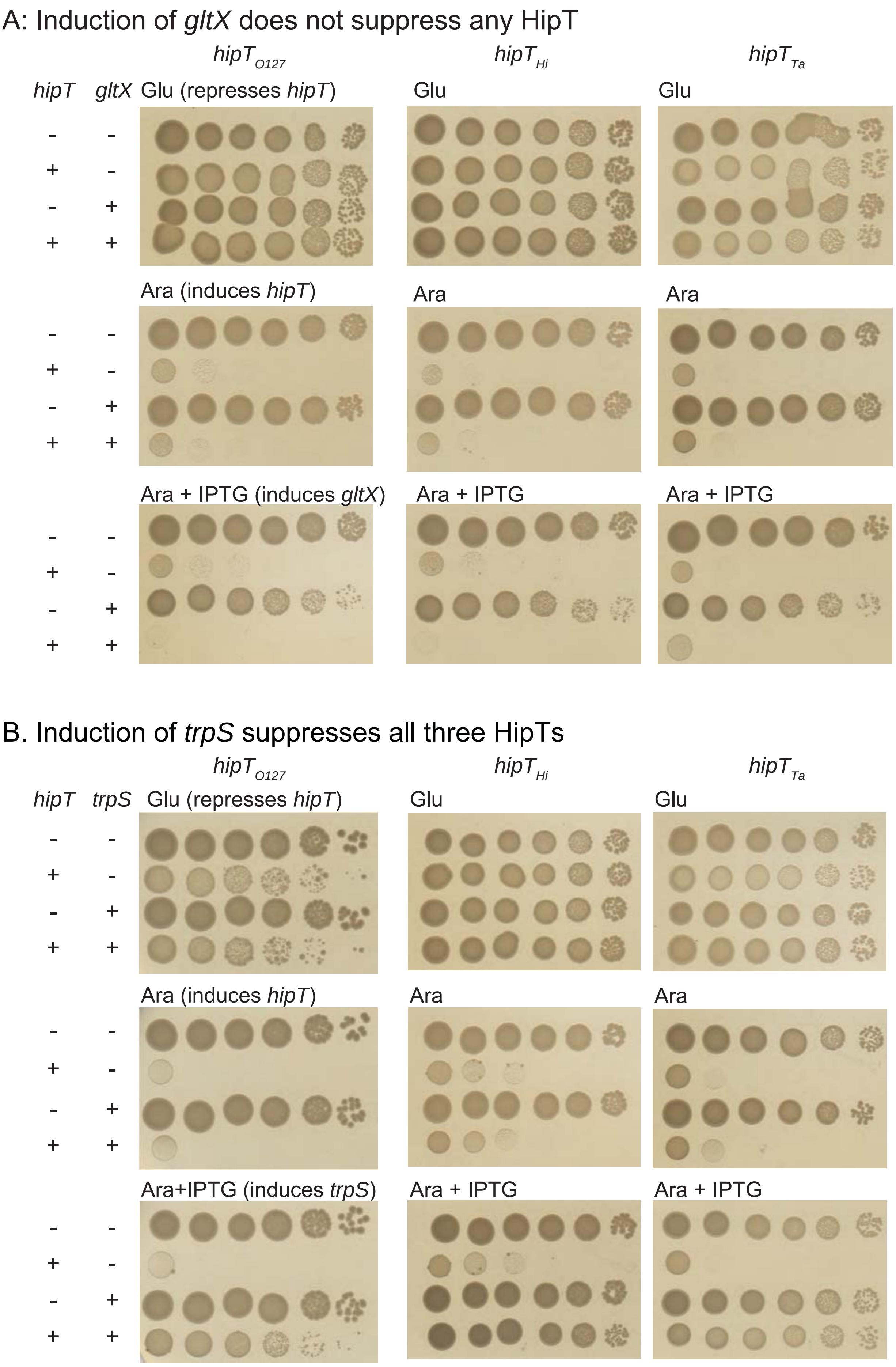
A: Induction of gltX does not suppress any HipT
hipT O127
hipT Hi
hipT Ta
hipT
gltX
Glu (represses hipT)
Glu
Glu
Ara (induces hipT)
Ara
Ara
Ara + IPTG (induces gltX)
Ara + IPTG
Ara + IPTG
B. Induction of trpS suppresses all three HipTs
hipT O127
hipT Hi
hipT Ta
hipT
trpS
Glu (represses hipT)
Glu
Glu
Ara (induces hipT)
Ara
Ara
Ara+IPTG (induces trpS)
Ara + IPTG
Ara + IPTG

Figure S5

Figure S5. Overproduction of TrpS suppresses $HipT_{O127}$, $HipT_{Hi}$ and $HipT_{Ta}$-mediated growth-inhibition whereas GltX has no such effect.

(**A**) Overnight cultures of MG1655 harboring (**left panel**) pSVN1 (pBAD33::*hipT*$_{O127}$), (**middle panel**) pSVN135 (pBAD33::*hipT*$_{Hi}$) or (**right panel**) pSVN129 (pBAD33::*hipT*$_{Ta}$) or the empty pBAD33 vector combined with pEG::*gltX* (pMG25::*gltX*) or the empty high-copy-number vector pMG25, as indicated, were diluted to similar OD_{600} values and washed in PBS before 10 times dilution series were spotted on LBA-plates containing the appropriate antibiotics and glucose (0.2%), arabinose (0.2%) or arabinose (0.2%) plus IPTG (200 μM), respectively.

(**B**) Overnight cultures of MG1655 harboring (**left panel**) pSVN1 (pBAD33::*hipT*$_{O127}$), (**middle panel**) pSVN135 (pBAD33::*hipT*$_{Hi}$) or (**right panel**) pSVN129 (pBAD33::*hipT*$_{Ta}$) or the empty pBAD33 vector combined with pSVN103 (pNDM220::*trpS*) or the empty low-copy-number vector pNDM220, as indicated, were diluted to similar OD_{600} values and washed in PBS before 10 times dilution series were spotted on LBA-plates containing the appropriate antibiotics and glucose (0.2%), arabinose (0.2%) or arabinose (0.2%) plus IPTG (200 μM), respectively.
