# Supplementary material for: Serine-Threonine Kinases Encoded by Split hipA Homologs Inhibit Tryptophanyl-tRNA Synthetase
Source: mBio. 2019 Jun 18;10(3):e01138-19. doi: 10.1128/mBio.01138-19 (PMC6581861; doi:10.1128/mBio.01138-19)
Supplement: FIG S6 [file mBio.01138-19-sf006.pdf]

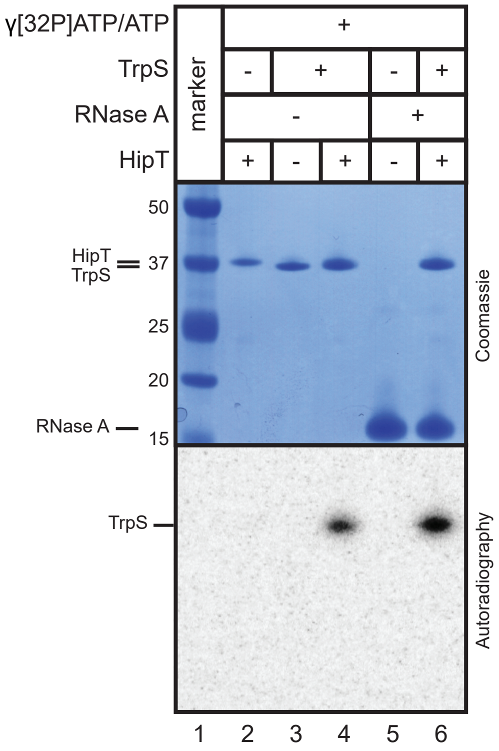

Figure S6

Figure S6. tRNA is not required for *in vitro* phosphorylation of TrpS by HipT$_{O127}$. Purified TrpS (1 µM; purified from BL21/ pSVN46), HipT$_{O127}$ (0.5 µM) (purified from BL21/pSVN42) and RNase A (0.1 mg/ml) were mixed with 0.1 µM γ[^{32}P]ATP and 66 uM ATP as indicated. Samples were treated as described in *Materials and methods*.
