# Supplementary material for: Serine-Threonine Kinases Encoded by Split hipA Homologs Inhibit Tryptophanyl-tRNA Synthetase
Source: mBio. 2019 Jun 18;10(3):e01138-19. doi: 10.1128/mBio.01138-19 (PMC6581861; doi:10.1128/mBio.01138-19)
Supplement: FIG S7 [file mBio.01138-19-sf007.pdf]

```
GltX     (1) -MKIKTRFAPSPTGYLHVGGARTALYSWLFARNHGGEFVLRIEDTDLERS
TrpS     (1) MTKPIVFSGAQPSGELTIGNYMGALRQWVNMQDD-YHCIYCIVDQHAITV

GltX    (50) TPEAIEAIMDGMNWLSLEWDEGPYYQTKRFDRYNAVIDQMLEEGTAYKCY
TrpS    (50) RQDAQKLRKATLDTLALYLACGIDPE-KSTIFVQSHVPEHAQLGWALNCY

GltX   (100) CSKERLEALREEQMAKGEKPRYDGRCRHSHEHHADDEPCVVRFANPQEGS
TrpS    (99) TYFGELSRMTQ---------------------------------------

GltX   (150) VVFDDQIRGPIEFSNQELDDLIIRRTDGSPTYNFCVVVDDWDMEITHVIR
TrpS   (110) --FKDKSARYAENINAGLFDYPVLMAADILLYQTNLVPVGEDQKQHLELS
                                                        P at S239
GltX   (200) GEDHINNTPRQINILKALKAPVPVYAHVSMINGDDGKKLSKRHG-AVSVM
TrpS   (158) RDIAQRFNALYGEIFKVPEPFIPKSGARVMSLLEPTKKMSKSDDNRNNVI
                                                        P at S197
GltX   (249) QYRDDGYLPEALLNYLVRLGWSHGDQEIFTREEMIKYFTLNAVSKSASAF
TrpS   (208) GLLED---PKSVVKKIKRAVTDSDEPPVVR---------YDVQNKAGVSN

GltX   (299) NTDKLLWLNHHYINALPPEYVATHLQWHIEQENIDTRNGPQLADLVKLLG
TrpS   (246) LLDILSAVTGQSIPELEKQFEG-KMYGHLKGEVADAVSG-----MLTELQ

GltX   (349) ERCKTLKEMAQSCRYFYEDFAEFDADAAKKHLRPVARQPLEVVRDKLAAI
TrpS   (290) ERYHRFRNDEAFLQQVMKDGAEKASAHASRTLKAVYEAIGFVAKP-----

GltX   (399) TDWTAENVHHAIQATADELEVGMGKVGMPLRVAVTGAGQSPALDVTVHAI
TrpS   (335) --------------------------------------------------

GltX   (449) GKTRSIERINKALDFIAERENQQ
TrpS   (335) -----------------------
```

Figure S7

Figure S7. Sequence alignment of GltX and TrpS.
The alignment shows that S239 in GltX aligns to S197 in TrpS in the conserved sequence motifs KKLSKR and KKMSKS in GltX and TrpS, respectively.
