# Supplementary material for: Serine-Threonine Kinases Encoded by Split hipA Homologs Inhibit Tryptophanyl-tRNA Synthetase
Source: mBio. 2019 Jun 18;10(3):e01138-19. doi: 10.1128/mBio.01138-19 (PMC6581861; doi:10.1128/mBio.01138-19)
Supplement: FIG S8 [file mBio.01138-19-sf008.pdf]

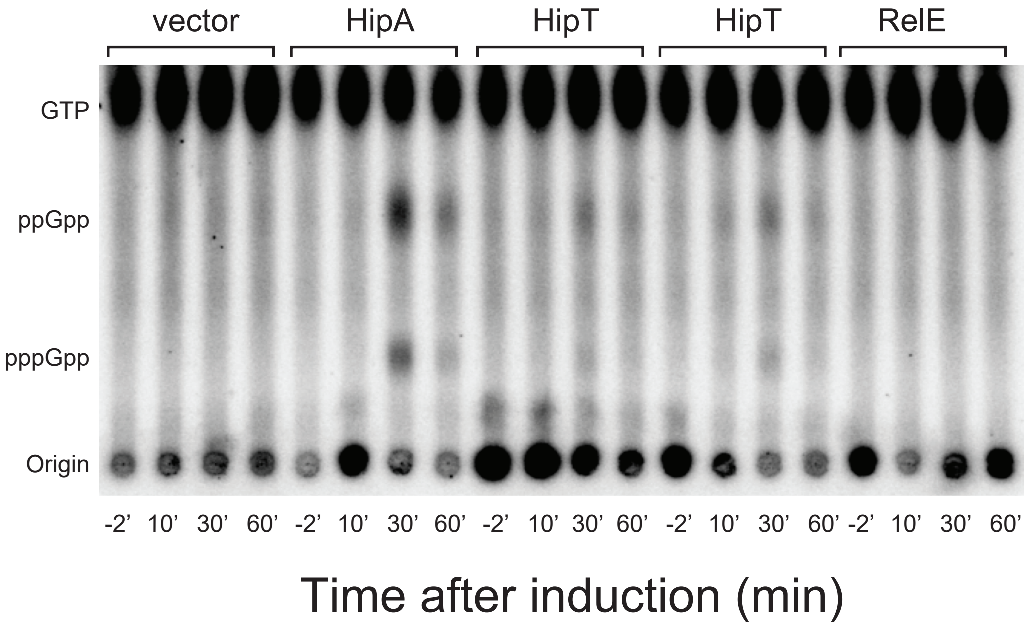

Figure S8

Figure S8. (p)ppGpp accumulation following overproduction of HipA, HipT$_{O127}$ and RelE. The Figure shows a repetition of the experiment shown in Fig. 4A. Cells of MG1655 carrying pAH1 (pNDM220::*hipA*), pSVN116 (pNDM220::*hipT$_{O127}$*) or pAH2 (pNDM220::*relE*) were grown exponentially in low phosphate MOPS minimal medium containing $H^{32}PO_4$. Samples were withdrawn before, 10, 30, and 60 minutes after induction of the toxin genes by the addition of IPTG (1 mM), analysed by TLC and phosphor imaging. The data contributed to the quantification shown in Fig. 4B. *Materials and methods* yield additional experimental details.
