# Supplementary material for: Serine-Threonine Kinases Encoded by Split hipA Homologs Inhibit Tryptophanyl-tRNA Synthetase
Source: mBio. 2019 Jun 18;10(3):e01138-19. doi: 10.1128/mBio.01138-19 (PMC6581861; doi:10.1128/mBio.01138-19)
Supplement: FIG S9 [file mBio.01138-19-sf009.pdf]

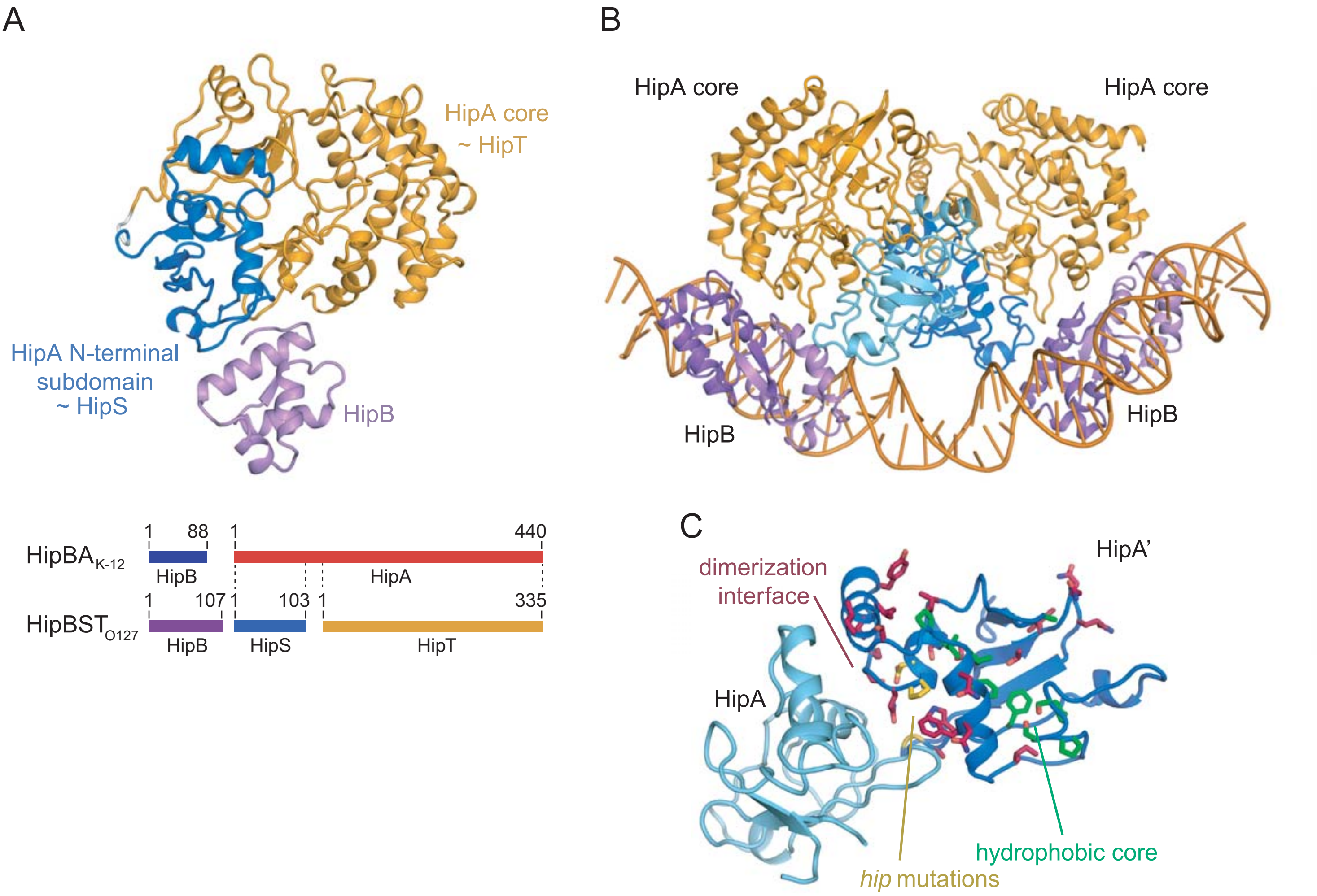

Figure S9

Figure S9. Modelling of the HipBST$_{O127}$ complex structure.
(**A**). Top; the crystal structure of *E. coli* HipBA (PDB ID: 4YG7) colored according to a sequence alignment with HipBST$_{O127}$, with the part corresponding to HipB$_{O127}$ in purple, HipS$_{O127}$ in blue, and HipT$_{O127}$ in yellow. Bottom; schematic overview of the sequence alignment with colors as above. HipA of *E. coli* K-12 is shown in red.
(**B**) The structure of HipBA bound to DNA shows that the part corresponding to HipS$_{O127}$ (the HipA N-terminal subdomain) mediates complex dimerization in the DNA-bound form.
(**C**) The N-terminal subdomain of HipA contains a hydrophobic core conserved in HipS$_{O127}$ (green) but a divergent dimerization interface (red). Two high-persister mutations in the N-subdomain-1 of HipA conserved among HipS orthologs are shown in yellow.
