# Supplementary material for: Serine-Threonine Kinases Encoded by Split hipA Homologs Inhibit Tryptophanyl-tRNA Synthetase
Source: mBio. 2019 Jun 18;10(3):e01138-19. doi: 10.1128/mBio.01138-19 (PMC6581861; doi:10.1128/mBio.01138-19)
Supplement: FIG S10 [file mBio.01138-19-sf010.pdf]

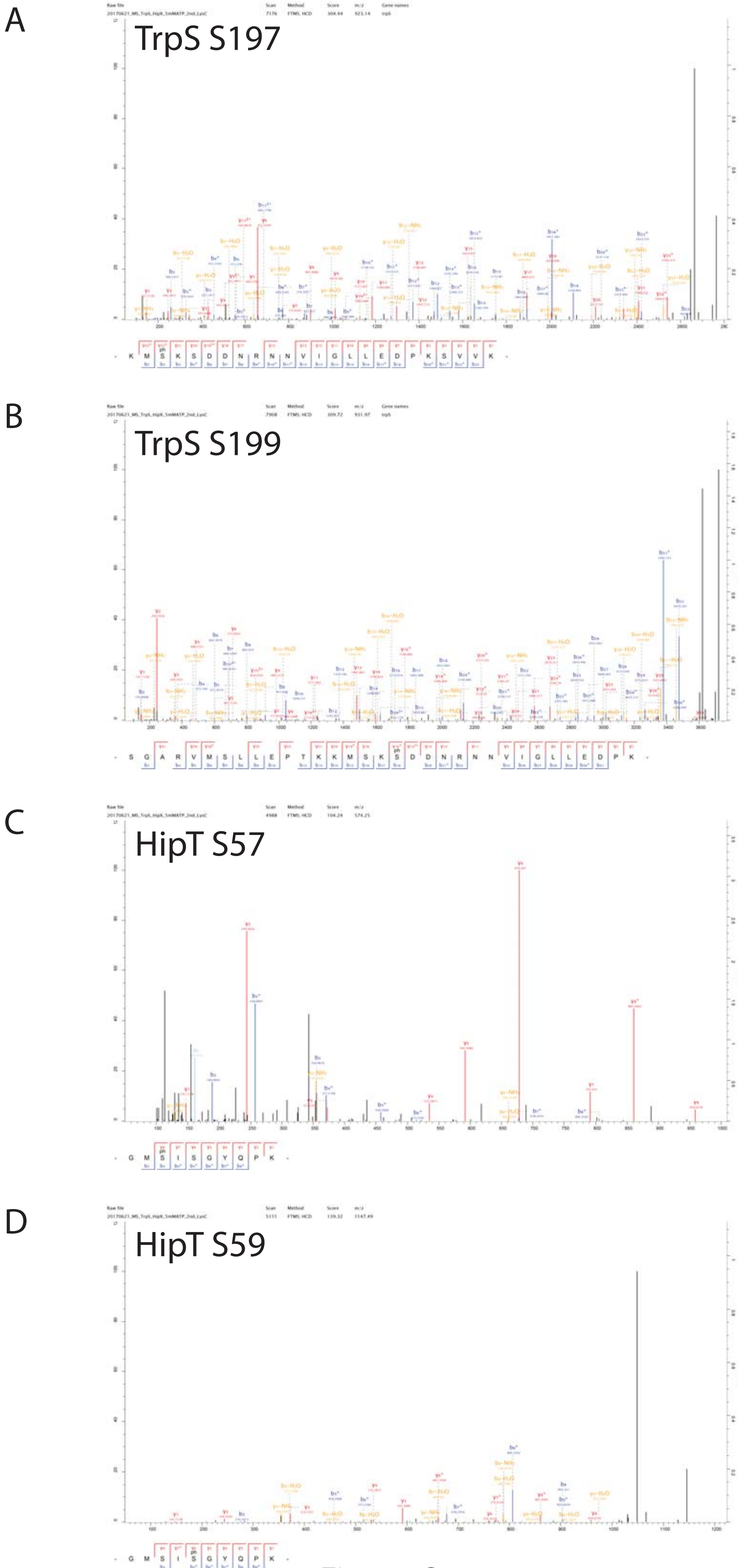

Figure S10

Figure S10. Representative mass spectra after *in vitro* kinase assay revealing HipT$_{O127}$-mediated phosphorylation sites on TrpS (A, B) and auto-phosphorylation sites of HipT$_{O127}$ (C, D).
